# Efficient sampling-based Bayesian Active Learning for synaptic characterization

**DOI:** 10.1371/journal.pcbi.1011342

**Published:** 2023-08-21

**Authors:** Camille Gontier, Simone Carlo Surace, Igor Delvendahl, Martin Müller, Jean-Pascal Pfister

**Affiliations:** 1 Department of Physiology, University of Bern, Bern, Switzerland; 2 Rehab Neural Engineering Labs, University of Pittsburgh, Pittsburgh, Pennsylvania, United States of America; 3 Department of Molecular Life Sciences, University of Zurich, Zurich, Switzerland; 4 Neuroscience Center Zurich, University of Zurich, Zurich, Switzerland; 5 University Research Priority Program (URPP), Adaptive Brain Circuits in Development and Learning (AdaBD), University of Zurich, Zurich, Switzerland; Durham University, UNITED KINGDOM

## Abstract

Bayesian Active Learning (BAL) is an efficient framework for learning the parameters of a model, in which input stimuli are selected to maximize the mutual information between the observations and the unknown parameters. However, the applicability of BAL to experiments is limited as it requires performing high-dimensional integrations and optimizations in real time. Current methods are either too time consuming, or only applicable to specific models. Here, we propose an Efficient Sampling-Based Bayesian Active Learning (ESB-BAL) framework, which is efficient enough to be used in real-time biological experiments. We apply our method to the problem of estimating the parameters of a chemical synapse from the postsynaptic responses to evoked presynaptic action potentials. Using synthetic data and synaptic whole-cell patch-clamp recordings, we show that our method can improve the precision of model-based inferences, thereby paving the way towards more systematic and efficient experimental designs in physiology.

## Author summary

Optimizing the design of an experiment is a critical problem in biology. However, most experiments still rely on suboptimal designs, which may not yield sufficient information about the studied system. Consequently, such experiments often require more observations to reach a certain result. An efficient theoretical framework to alleviate this issue is called Optimal Experiment Design (OED), in which experimental protocols are selected to reduce the uncertainty of inferred parameters. However, the applicability of OED methods to actual experiments is limited: they often require computations which are too long for sequential experiments, and do not generalize to different models. Here, we developed a method called Efficient Sampling-Based Bayesian Active Learning (ESB-BAL), and apply it to the problem of estimating the parameters of a chemical synapse from its evoked postsynaptic currents. After each new observation, the optimal next stimulation time can be computed using ESB-BAL. Using recordings in cerebellar brain slices, we show that our method is fast enough to be used in real-time biological experiments and can significantly reduce the uncertainty of inferred parameters. Our method can be readily used by experimentalists via a simple interface. Moreover, our proposed solution is general enough to be applicable to different experimental settings.

## Introduction

In neuroscience, machine learning, and statistics, a central problem is that of inferring the parameters *θ* of a model M. For instance, in supervised learning, one may want to learn the parameters of a Deep Neural Network (DNN) so as to minimize the difference between its output and training labels; in this case, M represents the DNN to be trained, and *θ* represents its weights and biases. Similarly, in biology, the parameters of a system can be studied by fitting a biophysical model to recorded observations. In most cases, these parameters can be neither directly measured nor analytically computed, but can be inferred using the recorded outputs of the system *y* as a response to input stimuli *x*. In biology, the physical quantities of a system (e.g. an organ, a cell, or a synapse) can be estimated by deriving a generative biophysical model M of the system, and by fitting its parameters *θ* to the observed responses *y* to experimental inputs *x*. By computing the likelihood of the outputs given the inputs and the parameters *p*(*y*|*x*, *θ*), it is possible to obtain either a point-based estimate of the parameters such as the maximum likelihood parameters *θ*_ML_ or the maximum a posteriori parameters *θ*_MAP_ [1], or to compute the full posterior distribution *p*(*θ*|*x*, *y*) ∝ *p*(*y*|*x*, *θ*) *p*(*θ*) using for instance the Metropolis-Hastings (MH) algorithm [2].

However, the accuracy of these estimates critically depends on the pair (*x*, *y*), and especially on how the successive input stimuli *x* = *x*_1:*T*_ are chosen. For instance, training a DNN on non independent and identically distributed (i.i.d.) training examples (i.e. blocked training) will lead to catastrophic forgetting [3]. On the other hand, most experiments in biology still rely on pre-defined and non-adaptive inputs *x*_1:*T*_, which may not yield sufficient information about the true parameters of the studied system. Consequently, experiments often require more observations or repetitions to reach a certain result, which increases their cost, time, and need for subjects.

**Fig 1:**
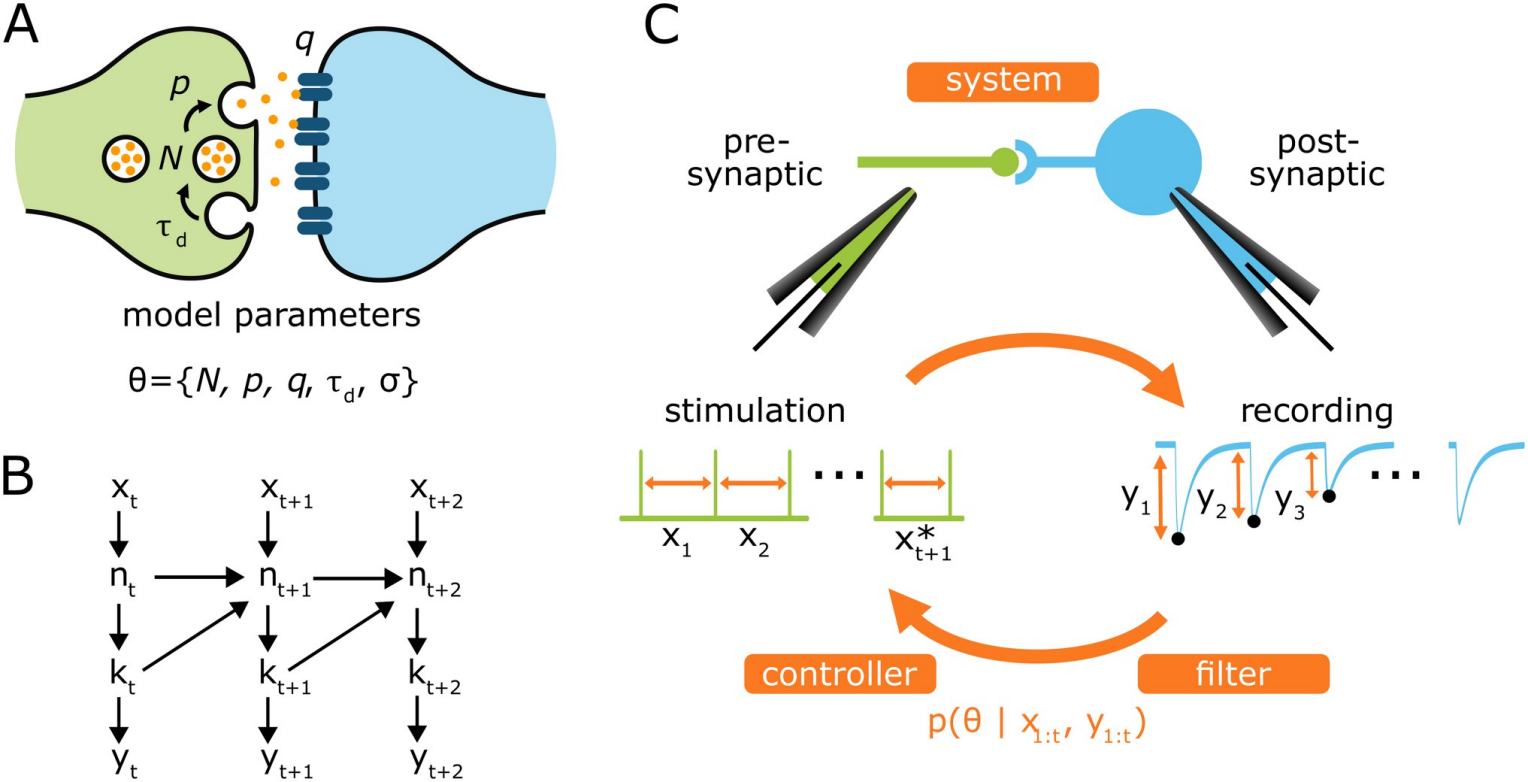
A: Model of binomial synapse with STD. In chemical synapses, the presynaptic terminal is characterized by the presence of *N* vesicles containing the neurotransmitter molecules, *n*_*t*_ of them being in the readily-releasable state [23]. Upon the arrival of a presynaptic spike, these vesicles will stochastically fuse with the plasma membrane and release their neurotransmitters into the synaptic cleft. After spike *t*, *k*_*t*_ vesicles (out of the *n*_*t*_ available ones in the readily-releasable pool) release their neurotransmitters with a probability *p*. Neurotransmitters will bind to postsynaptic receptors: a single release event triggers a quantal response *q*. The total recorded postsynaptic current *y*_*t*_ (i.e. the output of the system) is the sum of the effects of the *k*_*t*_ release events. After releasing, vesicles are replenished with a certain time constant *τ*_*D*_, which determines short-term depression. B: Modelisation of the synapse an an IO-HMM [10]. C: Bayesian Active Learning applied to biology. At each time step, the response of the **system** (e.g. here a synapse) to artificial stimulation is recorded. This observation *y*_*t*_ is used by the **filter** to compute the posterior distribution of parameters *p*(*θ*|*x*_1:*t*_, *y*_1:*t*_). Given this posterior, the **controller** then computes the next input $x_{t+1}^{*}$ to maximize the expected gain of information of the next observation. In classical experiment design, the inputs *x*_1:*T*_ are defined and fixed prior to the recordings.

An efficient framework to alleviate this issue is called Bayesian Active Learning (BAL). Knowing the current estimate of the parameters, the experimental protocol (i.e. the next input *x*_*t*+1_) can be optimized on the fly to maximize the mutual information between the recordings and the parameters (Fig 1C). BAL is a branch of Optimal Experiment Design (OED) theory [4–6]. It has already been used in neuroscience to infer the parameters of a Generalized Linear Model (GLM) [7], the nonlinearity in a linear-nonlinear-Poisson (LNP) encoding model [8], the receptive field of a neuron [9], or the parameters of a Hidden Markov Model (HMM) [10].

However, implementing BAL for biological settings can be challenging, especially for real-time applications. Its applicability to real experiments is limited by two main drawbacks. Firstly, it requires computing an update of the posterior distribution of parameters after each time step, and using it to compute the expected information gain from future experiments. This involves solving an optimization problem over a possibly high-dimensional stimulus space: current methods are either too time consuming, or only applicable to specific models. Secondly, to reduce computational complexity, classical implementations of BAL usually only optimize for the immediate next stimulus input. This classical myopic approach disregards all future observations in the experiment, and is thus possibly sub-optimal [6, 11, 12].

Our main contribution is to provide a general framework for approximate online active learning, called Efficient Sampling-Based Bayesian Active Learning (ESB-BAL). We use particle filtering, which is a highly versatile filtering method [13] for posterior computation, and propose a parallel computing implementation [14, 15] for efficient posterior update and information computation. Whereas previous implementations of active learning either relied on time consuming Monte Carlo (MC) methods [16, 17] or were only applicable to special cases, such as linear models or GLM [7], our proposed solution is fast enough to be used in real-time biological experiments and can be applied to any state-space model.

To illustrate our method, we apply it to the problem of inferring the parameters of a chemical synapse with Short-Term Depression (STD). Upon the arrival of a presynaptic action potential, vesicles from a pool of *N* independent release sites will fuse with the presynaptic plasma membrane with a probability *p*, each of these release events giving rise to a quantal current *q* [18, 19]. In addition, synaptic transmission is also dynamic. Short-term depression occurs when the inter-stimulation interval (ISI) is shorter than the time needed for synaptic vesicle replenishment [20]. A synapse exhibiting STD can thus be described by its parameters *N*, *p*, *q*, and by its depression time constant. These parameters can be inferred using excitatory postsynaptic currents (EPSCs) recorded from the postsynaptic cell and elicited by stimulating the presynaptic axon. The accuracy of these estimates critically depends on the presynaptic stimulation times: if inter-stimulation intervals are longer than the depression time constant, STD will not be precisely quantified. But if the stimulation frequency is too high, the pool of presynaptic vesicles will be depleted, leading to poor parameter estimates [21, 22]. Synaptic characterization is thus a relevant example application for ESB-BAL, as it requires careful tuning of the inputs *x*_1:*T*_, which in this case correspond to the inter-stimulation intervals (i.e. input *x*_*t*_ is the time interval between stimulation numbers *t* − 1 and *t*); but it is also a challenging one: computation needs to be faster than the typical ISI, which can be on the order of a few milliseconds. Using synthetic data, we show that our method allows to significantly reduce the uncertainty of the estimate in comparison to classically used non-adaptive stimulation protocols. We also show that the rate of information gain (in bit/s) of the whole experiment can be optimized by adding a penalty term for longer ISIs. Lastly, we extend active learning to non-myopic designs. Using recordings from cerebellar mossy fiber to granule cell synapses from acute mouse brain slices, we show that our framework is sufficiently efficient for optimizing not only the immediate next stimulus, but rather the future stimuli in the experiment.

## Results

### A general setting for Bayesian Active Learning

When using active learning in sequential experiments, three key elements need to be defined (Fig 1C):

1. The **system** to be studied: it is described by a generative model M, which parameters *θ* can be inferred from its observed responses *y*_1:*T*_ to a set of *T* input stimuli *x*_1:*T*_. Given the stochastic nature of most systems studied in biology, the random variable *Y*_1:*T*_ corresponding to the observations can take various values *y*_1:*T*_ according to a distribution *p*(*y*_1:*T*_|*x*_1:*T*_, *θ*). In our application example of BAL, the system will be a model of binomial neurotransmitter release (see Section The system: A binomial model of neurotransmitter release).
2. A **filter** that computes the posterior distribution of the parameters given the previous inputs and observations *p*(*θ*|*x*_1:*t*_, *y*_1:*t*_): after each new input *x*_*t*+1_ and observation *y*_*t*+1_, it is updated to obtain *p*(*θ*|*x*_1:*t*+1_, *y*_1:*t*+1_) (see Section The filter: Online computation of the posterior distributions of parameters).
3. A **controller** that computes the next optimal input stimuli $x_{t+1}^{*}$ so as to maximize a certain utility function, which is often defined as the mutual information between the parameter random variable Θ and the response random variable *Y*_*t*+1_ given the experimental inputs $I_{x_{t+1}}\left(\Theta ;Y_{t+1}|h_{t}\right)$, where *h*_*t*_ = (*x*_1:*t*_, *y*_1:*t*_) is the experiment history (see Section The controller: Computation of the optimal next stimulation time).

Throughout the paper, we use upper-case notations for random variables, and lower-case notations for the specific values they might take (Table 1). For instance, Θ is the random variables corresponding to the hidden parameters of the system, while *θ* describes a specific value of these parameters. Similarly, *Y*_*t*_ is the random variable corresponding to the output of the system at time step *t*, while *y*_*t*_ is the actual value observed at time step *t*. Hence, *H*(Θ) will represent the entropy of the random variable Θ, while *p*(Θ = *θ*) is the probability of Θ taking the specific value *θ*. For simplicity, we will often use the shorter notation *p*(*θ*) for *p*(Θ = *θ*). This distinction is crucial e.g. in Eq 3, to distinguish the conditional entropy $H_{x_{t+1}}\left(\Theta |h_{t},Y_{t+1}\right)=\int dy_{t+1}p\left(y_{t+1}|h_{t},x_{t+1}\right)H_{x_{t+1}}\left(\Theta |h_{t},Y_{t+1}=y_{t+1}\right)$ from its value given a specific realization of the future observation $H_{x_{t+1}}\left(\Theta |h_{t},Y_{t+1}=y_{t+1}\right)$.

**Table 1:** Notations.

**Indices:**	
*t* ≤ *T*	Number of observations
*i* ≤ *M*_out_	Number of outer particles
*j* ≤ *M*_in_	Number of inner particles
**Parameters:**	
*N*	Number of presynaptic independent release sites [-]
*p*	Release probability upon the arrival of a presynaptic spike [-]
*q*	Quantum of postsynaptic current elicited by one release event [A]
*σ*	Standard deviation of the recording noise [A]
*τ*_*D*_	Time constant of synaptic vesicle replenishment [s]
**Random variables:**	
*θ*	Vector of unknown parameters
*Y*_*t*_	Output of the system at time *t*
**Functions:**	
*p*_*θ*_(⋅)	Probability distribution conditioned on Θ = *θ*
*I*(⋅;⋅)	Mutual information
*H*(⋅)	Differential entropy
**Others:**	
*x*_*t*_	Input to the system at time step *t* (*t*^*th*^ inter-spike interval)
*y*_*t*_	Recording at time step *t* (*t*^*th*^ EPSC amplitude)
*h*_*t*_	History of observations (*x*_1:*t*_, *y*_1:*t*_)
M	Generative model of the studied system
*n*_*t*_	Number of vesicles in the readily-releasable state immediately
before spike *t*
*k*_*t*_	Number of released vesicles after spike *t*

In synaptic characterization, inputs correspond to a set of *T* stimulation times *x*_1:*T*_ and observations correspond to recorded excitatory postsynaptic currents (EPSCs) *y*_1:*T*_. In case of successive experiments [9], the mutual information between the parameters and the next observation *Y*_*t*+1_ conditioned on the experiment history *h*_*t*_ = (*x*_1:*t*_, *y*_1:*t*_) is:

$$\begin{array}{c} I_{x_{t+1}}\left(\Theta ;Y_{t+1}|h_{t}\right)=H\left(\Theta |h_{t}\right)-H_{x_{t+1}}\left(\Theta |h_{t},Y_{t+1}\right) \end{array}$$ (1)

where *H*(Θ|*h*_*t*_) is the entropy of Θ given the experiment history up to time step *t*:

$$\begin{array}{c} H\left(\Theta |h_{t}\right)=-\int d\theta p\left(\theta |h_{t}\right)\text{log}\;p\left(\theta |h_{t}\right) \end{array}$$ (2)

and

$$\begin{array}{c} H_{x_{t+1}}\left(\Theta |h_{t},Y_{t+1}\right)=\int dy_{t+1}p\left(y_{t+1}|h_{t},x_{t+1}\right)H_{x_{t+1}}\left(\Theta |h_{t},Y_{t+1}=y_{t+1}\right) \end{array}$$ (3)

is the conditional entropy of Θ given the future observation random variable *Y*_*t*+1_. Since the actual value of the future observation is unknown, we take the average over *y*_*t*+1_ of the conditional entropy $H_{x_{t+1}}\left(\Theta |h_{,}Y_{t+1}=y_{t+1}\right)$ conditioned on a certain value *y*_*t*+1_. As the predictive distribution depends on the unknown parameters, we also have to take an average over *θ*, using the current posterior distribution *p*(*θ*|*h*_*t*_) at time *t* [7]:

$$\begin{array}{c} p\left(y_{t+1}|h_{t},x_{t+1}\right)=\int d\theta p\left(y_{t+1}|h_{t},x_{t+1},\theta \right)p\left(\theta |h_{t}\right) \end{array}$$ (4)

The goal of Bayesian active learning is to select the next stimulation to maximize the mutual information between the parameters and all future observations:

$$\begin{array}{c} x_{t+1}^{*}=\underset{x_{t+1}\in S_{t+1}}{\text{arg}\;\text{max}}\;\underset{n}{\text{max}}\;\underset{x_{t+2:t+n}\in S_{t+2:t+n}}{\text{max}}I_{x_{t+1:t+n}}\left(\Theta ;Y_{t+1:t+n}|h_{t}\right) \end{array}$$ (5)

where $S_{t+1}$ is the set of possible inputs at time step *t* + 1 and $S_{t+2:t+n}$ is the set of possible protocols for the stimulations from time step *t* + 2 to *t* + *n*. This set of protocols includes all the stimulation constraints, e.g. the remaining time of the experiment or the minimal inter-stimulation time. Optimizing all future inputs is an intractable problem (especially for online applications), since the algorithmic complexity scales exponentially with the number of observations *n*. For this reason, BAL only optimizes for the next stimulus (an approach referred to as a *myopic* design) (see Fig 1C):

$$\begin{array}{c} x_{t+1}^{*}=\underset{x_{t+1}\in S_{t+1}}{\text{arg max}}\;I_{x_{t+1}}\left(\Theta ;Y_{t+1}|h_{t}\right) \end{array}$$ (6)

Different methods have been proposed to compute Eq 6. Monte Carlo (MC) methods [16] or a variational approach [17] can be employed, but they usually require long computation times that can be impractical if the time between successive experiments is short. Closed-form solutions or approximations can be computed only for some special cases, such as linear models or GLM [7].

### The system: A binomial model of neurotransmitter release

To illustrate our ESB-BAL framework, we apply it to the problem of estimating the parameters of a chemical synapse, represented as a state-space model with unobservable hidden states and input-dependent state transitions. A classical used model to describe the release of neurotransmitters at chemical synapses is called the binomial model [1, 2, 19–21, 24, 25]. According to this model, a synapse is described as an Input-Output Hidden Markov Model (IO-HMM [10]) with the following parameters (units are given in square brackets, see also Fig 1A):

- *N* (the number of presynaptic independent release sites [-]);
- *p* (their release probability upon the arrival of a presynaptic spike [-]);
- *q* (the quantum of current elicited in the postsynaptic cell by one release event [A]);
- *σ* (the standard deviation of the recording noise [A]);
- *τ*_*D*_ (the time constant of synaptic vesicle replenishment [s]).

The variables *n*_*t*_ and *k*_*t*_ represent, respectively, the number of available vesicles in the readily-releasable state at the moment of spike *t* (with 0 ≤ *n*_*t*_ ≤ *N*), and the number of vesicles (among *n*_*t*_) released after spike *t* (with 0 ≤ *k*_*t*_ ≤ *n*_*t*_). For simplicity, we use the notations *p*_*θ*_(⋅) = *p*(⋅|*θ*) with *θ* = [*N*, *p*, *q*, *σ*, *τ*_*D*_], and *z*_*t*_ ≔ (*n*_*t*_, *k*_*t*_) to refer to the hidden variables at time step *t*.

To summarize, the synapse is modelled as an IO-HMM, where:

- The input *x*_*t*_ refers to the time interval since the previous stimulation, i.e., to the inter-spike interval;
- The hidden state variable *z*_*t*_ ≔ (*n*_*t*_, *k*_*t*_) encompasses both the number of available vesicles immediately before spike *t* (*n*_*t*_ ∈ {0, …, *N*}) and the corresponding number of released vesicles (*k*_*t*_ ∈ {0, …, *n*_*t*_});
- The observable variable *y*_*t*_ is the recorded value of the postsynaptic current due to spike *t*. De facto, the postsynaptic current is continuously monitored: the specific value *y*_*t*_ is computed as the peak amplitude following spike *t* (see Materials and methods).

The probability of recording a set of *T* EPSCs *p*_*θ*_(*y*_1:*T*_) is computed as the marginal of the joint distribution of the observations *y*_1:*T*_ and the hidden variables *z*_1:*T*_, i.e. $p_{\theta }\left(y_{1:T}\right)=\sum_{z_{1:T}}p_{\theta }\left(y_{1:T},z_{1:T}\right)$, where the joint distribution *p*_*θ*_(*y*_1:*T*_, *z*_1:*T*_) = *p*_*θ*_(*y*_1:*T*_, *n*_1:*T*_, *k*_1:*T*_) is given by

$$\begin{array}{c} p_{\theta }\left(y_{1:T},n_{1:T},k_{1:T}\right)=p_{\theta }\left(y_{1}|k_{1}\right)p_{\theta }\left(k_{1}|n_{1}\right)p_{\theta }\left(n_{1}\right)\prod_{t=2}^{T}p_{\theta }\left(y_{t}|k_{t}\right)p_{\theta }\left(k_{t}|n_{t}\right)p_{\theta }\left(n_{t}|n_{t-1},k_{t-1},x_{t}\right) \end{array}$$ (7)

where

$$\begin{array}{c} p_{\theta }\left(y_{t}|k_{t}\right)=N\left(y_{t};qk_{t},\sigma^{2}\right) \end{array}$$ (8)

is the emission probability, i.e. the probability to record output *y*_*t*_ knowing that *k*_*t*_ vesicles released neurotransmitter; *p*_*θ*_(*k*_*t*_|*n*_*t*_) is the binomial distribution and represents the probability that, given *n*_*t*_ available vesicles, *k*_*t*_ of them will indeed release neurotransmitter:

$$\begin{array}{c} p_{\theta }\left(k_{t}|n_{t}\right)=(\begin{array}{c} n_{t}\\ k_{t} \end{array})p^{k_{t}}{\left(1-p\right)}^{n_{t}-k_{t}} \end{array}$$ (9)

Finally, *p*_*θ*_(*n*_*t*_|*n*_*t*−1_, *k*_*t*−1_, *x*_*t*_) represents the process of vesicle replenishment. During the time interval *x*_*t*_, each empty vesicle can refill with a probability $\pi \left(x_{t}\right)=1-\text{exp}(-\frac{x_{t}}{\tau_{D}})$ such that the transition probability *p*_*θ*_(*n*_*t*_|*n*_*t*−1_, *k*_*t*−1_, *x*_*t*_) is given by:

$$\begin{array}{c} p_{\theta }\left(n_{t}|n_{t-1},k_{t-1},x_{t}\right)=(\begin{array}{c} N-n_{t-1}+k_{t-1}\\ n_{t}-n_{t-1}+k_{t-1} \end{array})\pi {\left(x_{t}\right)}^{n_{t}-n_{t-1}+k_{t-1}}{\left(1-\pi \left(x_{t}\right)\right)}^{N-n_{t}} \end{array}$$ (10)

One can note that *n*_*t*_ = *n*_*t*−1_ − *k*_*t*−1_ + *v*_*t*_, where *v*_*t*_ ∼ Bin(*N* − *n*_*t*−1_ + *k*_*t*−1_, *π*(*x*_*t*_)) is the number of refilled vesicles during the time interval *x*_*t*_. Eqs 7 to 10 define the observation model of the studied system (see Fig 1), i.e. the probability of a set of observations *y*_1:*T*_ given a vector of stimuli *x*_1:*T*_ and a vector of parameters *θ*.

### The filter: Online computation of the posterior distributions of parameters

To be applicable for online experiments, the filtering block, which will compute the posterior distribution of parameters *p*(*θ*|*h*_*t*_), needs to satisfy two requirements:

1. It must be sufficiently versatile to be applied to different systems and models;
2. It must be online (i.e. its algorithmic complexity should not increase with the number of observations) [26].

A promising solution is particle filtering [27], and especially the Nested Particle Filter (NPF) [13]. This algorithm is asymptotically exact and purely recursive, thus allowing to directly estimate the parameters of a HMM as recordings are acquired.

The NPF relies on two nested layers of particles to approximate the posterior distributions of both the static parameters *θ* of the model and of its hidden states *z*_*t*_. A first outer filter with $M_{\text{out}}$ particles is used to compute the posterior distribution of parameters *p*(*θ*|*h*_*t*_), and for each of these particles, an inner filter with *M*_in_ particles is used to estimate the corresponding hidden states *z*_*t*_ (so that the total number of particles in the system is $M_{\text{out}}\times M_{\text{in}}$). After each new observation, these particles are resampled based on their respective likelihoods, hence updating their posterior distributions (S1 Fig).

The NPF was originally proposed for static HMMs, in which the state transition probability *p*(*z*_*t*+1_|*z*_*t*_, *θ*) is supposed to be constant. Here, we extend it to the more general class of Input-Output Hidden Markov Models (IO-HMMs, a subset of which is called GLM-HMMs in neuroscience, see [10]), in which the state transition probability at time step *t* depends on an external input *x*_*t*_. For instance, state transition in our model of synapse is not stationary, but depends on the ISI *x*_*t*_.

The filter (Algorithm 1) relies on the following approximation to recursively compute the likelihood of each particle. Once the observation *y*_*t*_ has been recorded, the likelihood of particle $\theta_{t}^{i}$, with $i\in \{1,...,M_{\text{out}}\}$, depends on

$$\begin{array}{c} p\left(\theta_{t}^{i}|y_{1:t}\right)\propto p\left(y_{t}|y_{1:t-1},\theta_{t}^{i},x_{t}\right)p\left(\theta_{t}^{i}|y_{1:t-1}\right) \end{array}$$ (11)

with

$$\begin{array}{c} p\left(y_{t}|y_{1:t-1},\theta_{t}^{i},x_{t}\right)=\sum_{z_{t-1:t}}\;p\left(y_{t}|z_{t},\theta_{t}^{i}\right)p\left(z_{t}|z_{t-1},\theta_{t}^{i},x_{t}\right)p\left(z_{t-1}|y_{1:t-1},\theta_{t}^{i}\right) \end{array}$$ (12)

If the variance of the jittering kernel *κ* (which mutates the samples to avoid particles degeneracy and local solutions, see Materials and methods) is sufficiently small, and hence if $\theta_{t}^{i}\approx \theta_{t-1}^{i}$, the approximation $p\left(z_{t-1}|y_{1:t-1},\theta_{t}^{i}\right)\approx p\left(z_{t-1}|y_{1:t-1},\theta_{t-1}^{i}\right)$ allows to approximate Eq 12 as $p\left(y_{t}|y_{1:t-1},\theta_{t}^{i},x_{t}\right)\approx \sum_{z_{t-1:t}}\;p\left(y_{t}|z_{t},\theta_{t}^{i}\right)p\left(z_{t}|z_{t-1},\theta_{t}^{i},x_{t}\right)p\left(z_{t-1}|y_{1:t-1},\theta_{t-1}^{i}\right)$, and hence to recursively compute Eq 11. In practice, the different terms in Eq 12 are computed as such: $p(y_{t}|z_{t},\theta_{t}^{i})$ corresponds to the *Likelihood* step of Algorithm 1; $p(z_{t}|z_{t-1},\theta_{t}^{i},x_{t})$ corresponds to the *Propagation* step; and $p(z_{t-1}|y_{1:t-1},\theta_{t}^{i})$ corresponds to the posterior distribution of hidden states at time *t* − 1 given the observations *y*_1:*t*−1_ up to *t* − 1.

Contrary to previous methods for fast posterior computation that were only applicable to specific models [7], our filter can be applied to any state-space dynamical system, including non-stationary and input-dependent ones. Moreover, it does not require to approximate the posterior as a Gaussian nor require a time consuming (and possibly unstable) numerical optimization step, while being highly parallelizable and efficient [14, 15].

**Algorithm 1**: Particle filtering for computing one step update of the posterior distribution of parameters

**Input**: ${\left\{\theta_{t-1}^{i}\right\}}_{1\leq i\leq M_{\text{out}}}$, ${\left\{n_{t-1}^{i,j},k_{t-1}^{i,j}\right\}}_{1\leq j\leq M_{\text{in}}}$, *x*_*t*_, *y*_*t*_;

**for** *i in* 1 … *M*_out_ **do**

 **Jittering**: update the outer particles $\theta_{t}^{i}=\kappa \left(\theta_{t-1}^{i}\right)$;

 **for** *j in* 1 … *M*_in_ **do**

  **Propagation**: Draw $n_{t}^{i,j}∼p\left(n_{t}^{i,j}|n_{t-1}^{i,j},k_{t-1}^{i,j},\theta_{t}^{i},x_{t}\right)$ and $k_{t}^{i,j}∼p\left(k_{t}^{i,j}|n_{t}^{i,j},\theta_{t}^{i}\right)$

  **Likelihood**: compute ${\tilde{w}}_{t}^{i,j}=p\left(y_{t}|n_{t}^{i,j},k_{t}^{i,j},\theta_{t}^{i}\right)$;

 **end**

 Compute $w_{t}^{i}=\frac{1}{M_{\text{in}}}\sum_{j}{\tilde{w}}_{t}^{i,j}$;

 **Normalization**: ${\tilde{w}}_{t}^{i,j}\leftarrow {\tilde{w}}_{t}^{i,j}/\left(M_{\text{in}}w_{t}^{i}\right)$;

 **Inner particles resampling**: resample ${\left\{n_{t}^{i,j},k_{t}^{i,j}\right\}}_{1\leq j\leq M_{\text{in}}}$ based on ${\left\{{\tilde{w}}_{t}^{i,j}\right\}}_{1\leq j\leq M_{\text{in}}}$;

**end**

**Normalization**: $w_{t}^{i}\leftarrow w_{t}^{i}/\sum_{i}w_{t}^{i^{\prime }}$;

**Outer particles resampling**: resample ${\left\{\theta_{t}^{i},{\left\{n_{t}^{i,j},k_{t}^{i,j}\right\}}_{1\leq j\leq M_{\text{in}}}\right\}}_{1\leq i\leq M_{\text{out}}}$ based on ${\left\{w_{t}^{i}\right\}}_{1\leq i\leq M_{\text{out}}}$;

**Output**: ${\left\{\theta_{t}^{i}\right\}}_{1\leq i\leq M_{\text{out}}}$, ${\left\{n_{t}^{i,j},k_{t}^{i,j}\right\}}_{1\leq j\leq M_{\text{in}}}$

### The controller: Computation of the optimal next stimulation time

The objective of experiment design optimization is to minimize the uncertainty of the estimates (classically quantified using the entropy) while reducing the cost of experimentation (defined as the number of required trials, samples, or observations). The optimal next stimulus $x_{t+1}^{*}$ that will maximize the mutual information (i.e. minimize the uncertainty about *θ* as measured by the entropy) can be written from Eqs 1, 3, and 6 as

$$\begin{array}{c} x_{t+1}^{*}=\underset{x_{t+1}\in S_{t+1}}{\text{arg min}}\int d\theta p\left(\theta |h_{t}\right)\int dy_{t+1}p\left(y_{t+1}|h_{t},x_{t+1},\theta \right)H_{x_{t+1}}\left(\Theta |h_{t},Y_{t+1}=y_{t+1}\right) \end{array}$$ (13)

Eq 13 requires to compute two (possibly high-dimensional) integrals over *θ* and *y*_*t*+1_, for which closed-form expressions only exist for specific models. To avoid long MC simulations, we propose to use mean-field computations and to replace integrals by point-based approximations. Firstly, instead of computing the full expectation over *p*(*θ*|*h*_*t*_), we set *θ* to its MAP value ${\hat{\theta }}_{t}$. Other estimators, such as the mean posterior value ${\hat{\theta }}_{t}=\int d\theta p\left(\theta |h_{t}\right)\theta$ (which can be conveniently approximated as ${\hat{\theta }}_{t}\approx \frac{1}{M_{\text{out}}}\sum_{i=1}^{M_{\text{out}}}\theta_{t}^{i}$), can be used. For parameters taking integer values, like *N*, this mean posterior can be rounded to the nearest value. Eq 13 thus becomes

$$\begin{array}{c} x_{t+1}^{*}\approx \underset{x_{t+1}\in S_{t+1}}{\text{arg min}}\int dy_{t+1}\;p\left(y_{t+1}|h_{t},x_{t+1},{\hat{\theta }}_{t}\right)H_{x_{t+1}}\left(\Theta |h_{t},Y_{t+1}=y_{t+1}\right) \end{array}$$ (14)

Depending on the nature of the studied system and on the time constraints of the experiment, different estimators can also be used, such as e.g. ${\hat{\theta }}_{t}={\text{arg max}}_{\theta }\;p\left(\theta |h_{t}\right)$. It is important to note that the posterior distribution *p*(*θ*|*h*_*t*_) is only reduced to a Dirac distribution *δ*(*θ* − *θ*_*t*_) here (in order to obtain a point-based estimate of *θ* and to simplify the computation of the integral over *y*_*t*+1_), but not for computing the computational entropy $H_{x_{t+1}}\left(\Theta |h_{t},Y_{t+1}=y_{t+1}\right)$ (see below). Secondly, instead of computing the full expectation over the future observation, we set *y*_*t*+1_ to its expected value; Eq 13 thus becomes

$$\begin{array}{c} x_{t+1}^{*}\approx \underset{x_{t+1}\in S_{t+1}}{\text{arg min}}\;H_{x_{t+1}}\left(\Theta |h_{t},Y_{t+1}=E\left(Y_{t+1}|h_{t},x_{t+1},{\hat{\theta }}_{t}\right)\right) \end{array}$$ (15)

In the general case, $E(Y_{t+1}|h_{t},x_{t+1},{\hat{\theta }}_{t})$ can be computed using Bayesian Quadrature [28]. More specifically, for our model of a chemical synapse, an analytical formulation for the expected value $E(Y_{t+1}|x_{1:t+1},{\hat{\theta }}_{t})$ can be efficiently derived using mean-field approximations (see Section Mean-field approximation of vesicle dynamics). For each candidate *x*_*t*+1_ in a given finite set $S_{t+1}$, the entropy $H(\Theta |h_{t},x_{t+1},Y_{t+1}=E\left(Y_{t+1}|h_{t},x_{t+1},{\hat{\theta }}_{t}\right))$ can be computed using Algorithm 1.

Finally, for computational efficiency reasons, instead of actually computing the posterior entropy, we are using an upper bound of this posterior entropy at any time step *t*, i.e. $\frac{1}{2}\;\text{log}\left|2\pi e\Sigma_{t}\right|$, where Σ_*t*_ is the covariance matrix of the particles ${\left\{\theta_{t}^{i}\right\}}_{1\leq i\leq M_{\text{out}}}$. Indeed, the maximum entropy distribution for a given covariance matrix Σ_*t*_ and a given mean *μ*_*t*_ is precisely the Gaussian distribution $N(\mu_{t},\Sigma_{t})$ for which the entropy is $\frac{1}{2}\;\text{log}\left|2\pi e\Sigma_{t}\right|$. It should be remembered that the in the limit of large amount of data, the posterior distribution will converge to a Gaussian distribution [29]. As a consequence the upper bound will be tight after a large number of observations. Note that this cost function has been previously used in Bayesian Active Learning [10]. Hence, the entropy in 15 is approximately minimized by minimizing the determinant of the covariance matrix of the particles drawn from $p(\Theta |h_{t},x_{t+1},Y_{t+1}=E\left(Y_{t+1}|h_{t},x_{t+1},{\hat{\theta }}_{t}\right))$:

$$\begin{array}{c} H_{x_{t+1}}\left(\Theta |h_{t},Y_{t+1}=E\left(Y_{t+1}|h_{t},x_{t+1},{\hat{\theta }}_{t}\right)\right)\approx \frac{1}{2}\text{log}\;\left|2\pi e\Sigma_{t+1}\right| \end{array}$$ (16)

### First setting: Reducing the uncertainty of estimates for a given number of observations

From the experimentalist point of view, a highly relevant question is how to optimize the stimulation protocol such that the measured EPSCs are most informative about synaptic parameters. Previous studies showed that some stimulation protocols are more informative than others, but ignored the temporal correlations of the number of readily-releasable vesicles [30] or did not compute which protocol would be most informative [1]. In classical deterministic experiment protocols, the stimulation times *x*_1:*T*_ are defined and fixed prior to the recordings. By contrast, active learning optimises the protocol on the fly as data are recorded.

**Fig 2:**
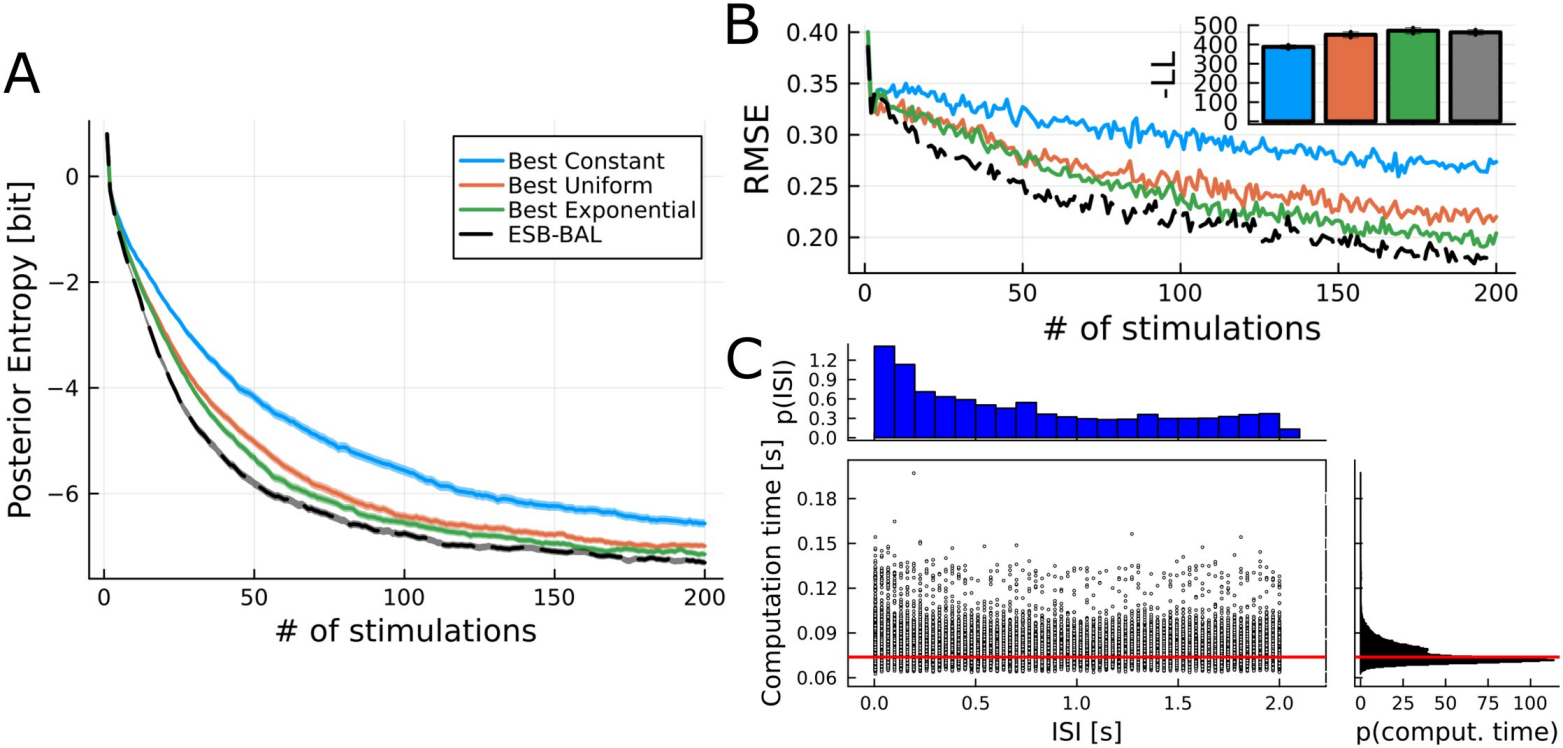
First setting: reducing the uncertainty of estimates for a given number of observations. A: Entropy of the posterior distribution of *θ* vs. number of observations for different stimulation protocols. Synthetic data were generated from a model of synapse with ground truth parameters N* = 7, p* = 0.6, q* = 1 pA, σ* = 0.2 pA, and $\tau_{D}^{*}=0.25$s [2]. Traces show average over 400 independent repetitions. Shaded area: standard error of the mean. B: RMSE for the same simulations. C: Histograms and scatter plot of the ISIs and the corresponding computation times for the ESB-BAL simulations. Note that the median computation time (horizontal red line) of 74*ms* corresponds to the time required to test 64 candidate intervals: hence, each tested interval takes approx. 1.16*ms*.

Results for a simulated experiment with ground-truth parameters N* = 7, p* = 0.6, q* = 1 pA, σ* = 0.2 pA, and $\tau_{D}^{*}=0.25$s (i.e. the same set of parameters θ* used in [2]) are displayed in Fig 2A. Here, we compare ESB-BAL to three deterministic protocols:

- in the *Constant* protocol, the synapse is probed at a constant frequency, i.e. *x*_t_ = *x*^cst^;
- in the *Uniform* protocol, ISIs are uniformly drawn from a set S of candidates *x*_*t*_ consisting of equidistantly separated values ranging from *x*^min^ = 0.005s (i.e. one order of magnitude shorter than the shortest ISI used in [1]) to *x*^max^, i.e. $x_{t}∼\text{Uniform}\left(\left[0.005,x^{\text{max}}\right]\right)$;
- finally, in the *Exponential* protocol, ISIs are drawn from an exponential distribution with mean *τ*. Such a protocol has been shown to provide better estimates of synaptic parameters compared to periodic spike trains with constant ISI [1, 30].

The efficiency of these deterministic protocols will depend on their respective parametrizations. To conservatively assess ESB-BAL, we optimize the values of *x*^cst^, *x*^max^, and *τ* so that the *Constant*, *Uniform*, and *Exponential* protocols have the best possible performance for the used ground-truth parameters θ* after 200 observations. S2 Fig shows the average final entropy decrease (i.e. the information gain) after 200 observations using the *Constant* (top), *Uniform* (middle), or *Exponential* (bottom) protocol, for different values of their hyperparameters. These deterministic protocols (with their optimal respective parametrizations) are then compared to ESB-BAL.

For the different protocols, the average (over 400 independent repetitions) differential entropy of the posterior distribution of parameters is plotted as a function of the number of observations (Fig 2A). ESB-BAL allows to reduce the uncertainty (as measured by the entropy) of the parameter estimates for a given number of observations. It should be noted that it is compared to deterministic protocols whose respective hyperparameters have been optimized offline, knowing the value of θ*. In real physiology experiments, classical protocols are non-adaptative and are defined using (possibly sub-optimal) default parameters. In contrast, in active learning the protocol is optimized on the fly as data are recorded, and its performance will not depend on a prior parametrization. Approximate optimal design via ESB-BAL thus outperforms the best possible *Constant*, *Uniform*, and *Exponential* protocols. Interestingly, it also outperforms a non-parametric optimized design (S3 Fig).

We also verify that ESB-BAL does not lead to biased estimates of *θ*, as its average RMSE outperforms that of other protocols (Fig 2B). Moreover, for each protocol, we assess whether estimated parameters provide a good description of held-out data. An estimate of the parameters ${\hat{\theta }}_{t}$ is obtained after *t* = 100 observations, and its likelihood is computed for observations up to *t* = 200. The inset in Fig 2B shows the mean negative log-likelihood of ${\hat{\theta }}_{t}$ for 50 repetitions of the protocols in Fig 2A: *Constant* (blue), *Uniform* (orange), *Exponential* (green), and ESB-BAL (grey). Results show no significant differences in the goodness of fit of estimated parameters for held-out data for the *Uniform*, *Exponential*, and ESB-BAL protocols. Interestingly, the *Constant* protocol yields a higher likelihood, but a higher RMSE, for estimated parameters. This means that, for this protocol, estimated parameters are a good fit to data, but that the said data are not informative enough to accurately infer the ground-truth values of the parameters.

Finally, we verify that ESB-BAL is sufficiently fast for online applications, as computation time exceeds the ISI in only a small proportion of cases (Fig 2C). Similar results can be observed for different sets of ground-truth parameters θ* (S4 Fig) or when only optimizing for the entropy of a specific parameter (S5 Fig).

**Fig 3:**
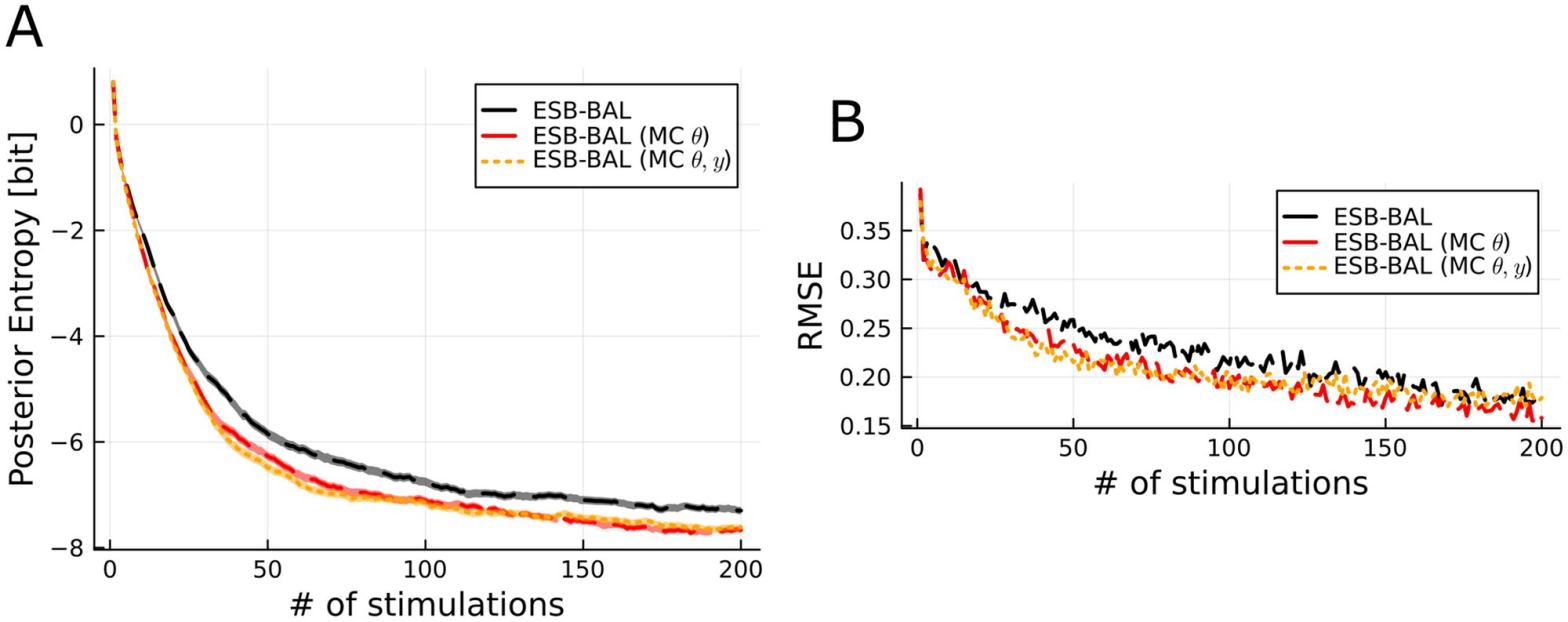
Assessing the effect of point-based approximations on the accuracy of ESB-BAL. Same setting as in Fig 2. In ESB-BAL (MC *θ*), the integral over *θ* in Eq 13 is computed using MC samples instead of the point-based approximation described in Eq 14. In ESB-BAL (MC *θ*, *y*), both integrals over *θ* and *y*_*t*+1_ in Eq 13 are computed using MC samples instead of the point-based approximations described in Eqs 14 and 15.

The computational efficiency of ESB-BAL is achieved through approximations in the computations required to implement the controller. To assess the effect of the sample approximations made in Eqs 14 and 15 on accuracy, we compared ESB-BAL to exact active learning, in which Eq 13 is computed exactly using MC samples (Fig 3). In ESB-BAL (MC *θ*), samples (which are used to compute the expectation over *θ*) are drawn from *p*(*θ*|*h*_*t*_), and corresponding point-based estimates of *y*_*t*+1_ are computed using Eq 23, as in Eq 15. Further, in ESB-BAL (MC *θ*, *y*) samples used to compute the expectation over *y*_*t*+1_ are drawn by randomly sampling from the inner particles (see Materials and methods). This shows that the approximations used in Algorithm 2 to make active learning online have only a small effect on performance.

### Second setting: Reducing the uncertainty of estimates for a given experiment time

**Fig 4:**
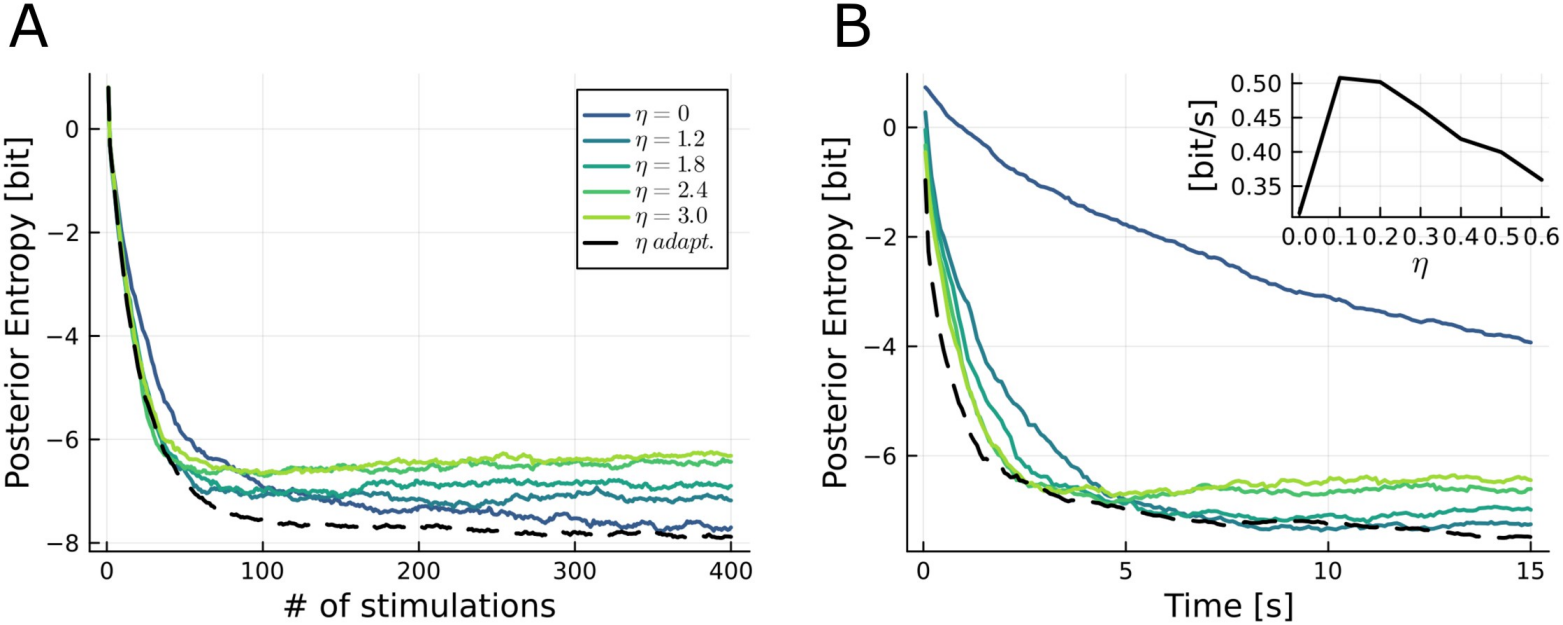
Second setting: reducing the uncertainty of estimates for a given experiment time (effect of penalizing long ISIs on parameter estimates uncertainty and rate of information gain). A: Posterior entropy *H*(Θ|*h*_*t*_) as a function of the stimulation number *t* for different values of *η* in Eq 18. Same settings as in Fig 2. B: Same results, but displayed as a function of time. Inset: average information rate (in bits/s) from *t* = 0 to *t* = 10s as a function of *η*. Results displayed for *α* = 0.05.

Active learning allows, for a given number of observations, to improve the reliability of the estimated parameters. However, in its classical implementation, only the next stimulus input is optimized, disregarding all future observations in the experiment (see the approximation made from Eqs 5 to 6). This myopic approach is thus sub-optimal. Moreover, neurophysiology experiments are not only constrained by the number of observations, but also by the total time of the experiment. Since cell viability and recording stability may be a limiting factor during an experiment, the total time of an experimental protocol $x_{\text{tot}}=\sum_{t=1}^{T}x_{t}$ also needs to be accounted for. Here, to account for the total time of the experiment, and to globally optimize the information gain per unit of time, we can go back to the mutual information expression in Eq 6 and use the chain rule to rewrite it as the following sum:

$$\begin{array}{c} I_{x_{t+1:t+n}}\left(\Theta ;Y_{t+1:t+n}|h_{t}\right)=I_{x_{t+1}}\left(\Theta ;Y_{t+1}|h_{t}\right)+\sum_{i=2}^{n}I_{x_{t+1:t+i}}\left(\Theta ;Y_{t+i}|Y_{t+1:t+i-1},h_{t}\right) \end{array}$$ (17)

where the first term on the r.h.s of Eq 17 is the “myopic term” that has been kept in Eq 6 while the second one is the “non-myopic term” and describes the information gain due to all the future events (from *t* + 2 to *t* + *n*), but still depends on *x*_*t*+1_. Computing this “non-myopic term” is computationally prohibitive. However, instead of simply ignoring it, we can approximate it. If we make the (rather strong) assumption that the future information gain is obtained at a constant rate *η* (in bits per seconds), then the information gain during the remaining time $x_{\text{tot}}-\sum_{i=1}^{t+1}x_{i}$ is given by $\eta \left(x_{\text{tot}}-\sum_{i=1}^{t+1}x_{i}\right)=-\eta x_{t+1}+c$ where the constant *c* is independent of *x*_*t*+1_. With this assumption, we can express the updated formulation of active learning (see original formulation in Eq 13) as

$$\begin{array}{c} x_{t+1}^{*(\eta )}=\underset{x_{t+1}\in S_{t+1}}{\text{arg min}}\;H_{x_{t+1}}\left(\Theta |h_{t},Y_{t+1}\right)+\eta x_{t+1} \end{array}$$ (18)

where *ηx*_*t*+1_ acts as penalty term for longer ISIs. The effect of the assumed future information rate *η* on the entropy of the posterior distribution of the parameters is displayed in Fig 4A. As expected, adding a penalty term to Eq 13 reduces the precision of the inferred parameter. The loss of information gain increases with the penalty weight *η*. However, increasing *η* also increases the speed of information gain, as seen in Fig 4B. Depending on the available time for the experiment, it is thus possible to tune *η* so as to find a trade-off between long-term precision (Fig 4A) and information rate (Fig 4B).

So far, we made the strong assumption that *η* is constant which is problematic for two reasons. Firstly, it is not accurate (the posterior entropy doesn’t decay linearly in time, as seen on Fig 4B) and secondly, it requires to chose *η* before the start of the experiment. To circumvent both issues at the same time, we implemented an adaptive estimation of the information rate:

$$\begin{array}{c} \eta_{t+1}=\alpha \frac{\Delta H_{t}}{x_{t}}+\left(1-\alpha \right)\eta_{t} \end{array}$$ (19)

where Δ*H*_*t*_ = *H*(Θ|*h*_*t*−1_) − *H*(Θ|*h*_*t*_) is the gain in entropy and *α* is the learning rate of the estimated information rate *η*_*t*_. As we can see in Fig 4, the adaptive estimated information rate provides a better performance than a fixed *η*.

### Third setting: Batch optimization and application to neural recordings

To reduce computational complexity, classical implementations of sequential experiment design usually only optimize for the immediate next observation, as in Figs 2, 3, and 4. However, it might be critical for some systems to optimize not only the next stimulus, but rather the next *n* stimuli of the experiment altogether (see Eq 5) [6, 12]. Synaptic characterization is a telling example: indeed, STD can only be observed for specifically organized batches of stimulation times, where the pool of presynaptic vesicles is first depleted by high-frequency stimulations and its refilling rate then probed using increasing ISIs. Moreover, it should be noted that, depending on the experimental set-up, optimizing batches of future stimuli is often simpler than optimizing each future stimulus. Indeed, some experimental apparatuses (such as the amplifiers that we used for synaptic stimulation), do not allow for online closed-loop input computation, but only accept programmed batches of inputs. Batch optimization thus allowed to circumvent hardware limitations.

When probing the presynaptic cell, neuroscientists usually use repetitions of a spike train consisting of a tetanic stimulation phase (sustained high-frequency stimulation used to deplete the presynaptic vesicles) followed by recovery spikes at increasing ISIs to probe the STD time constant [31]. These spike trains (especially the duration and frequency of the tetanic phase, and the ISI between recovery spikes) are usually not optimized, and are held constant throughout an entire experiment. Here, we show that ESB-BAL can be used to extend active learning to non-myopic designs (i.e. the optimization is not restricted to the timing of the next event, but takes into account the *n* next input simuli). Such an approach has already been proposed to enable the selection of maximally informative stimulus sequences, hence avoiding the drawbacks of selecting only one stimulus at a time [11].

**Fig 5:**
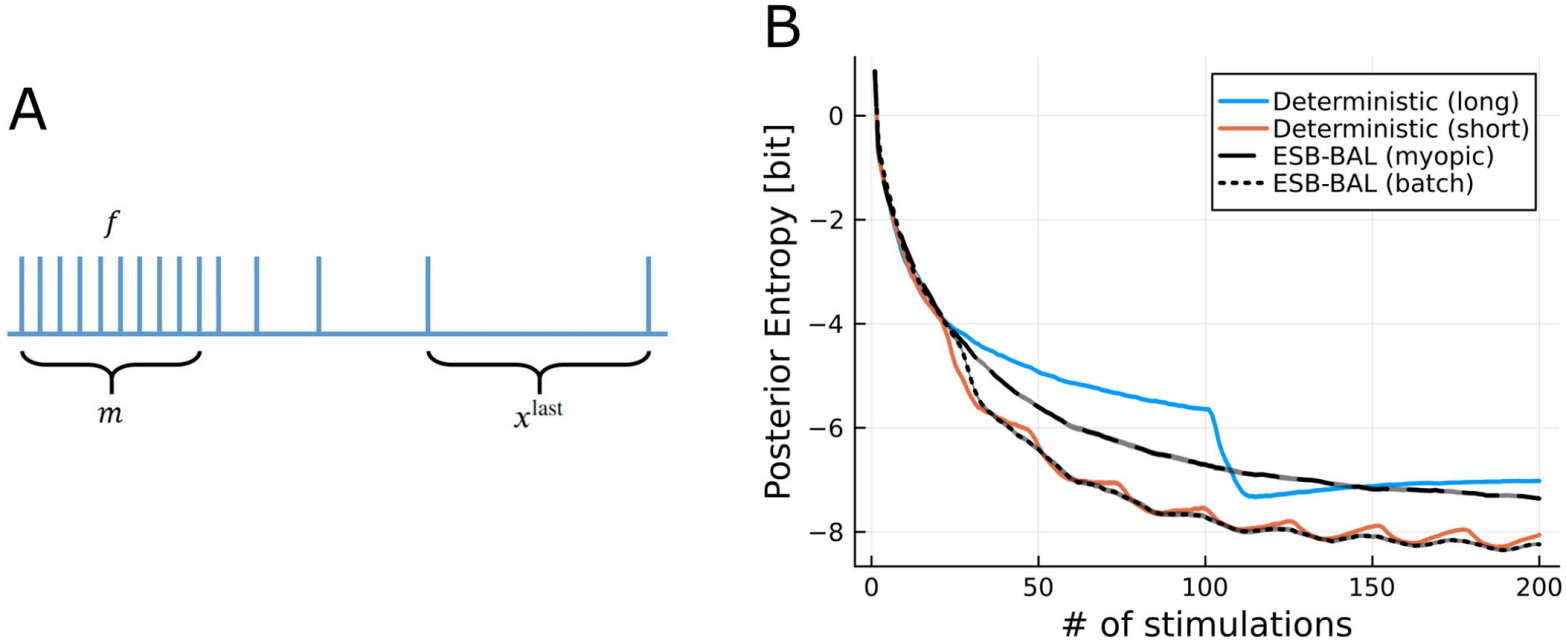
Third setting: batch optimization. A: Schematic of how elements in $S_{t+1:t+n}$ in Algorithm 3 are defined. They are chosen to span 3 parameters: the number *m* < *n* of spikes in the tetanic stimulation phase, the frequency *f* of spikes in the tetanic stimulation phase, and the duration of the final recovery ISI $x^{\text{last}}$. B: Simulated experiment with ground-truth parameters N* = 47, p* = 0.27, q* = 2.65 pA, σ* = 1.32 pA, and $\tau_{D}^{*}=0.17$s (i.e. the MAP values from one example cell studied in Fig 6B).

It should be stressed here that systematically searching over an *n*-dimensional space is computationally prohibitive when *n* is large. As a consequence, we restricted the optimisation to a low-dimensional subspace parametrised by 3 parameters $\left(m,f,x^{\text{last}}\right)$, see caption of Fig 5A for more details. Candidate batches are defined as such:

- *n* is the total number of stimulations;
- *m* < *n* is the number of spikes in the tetanic stimulation phase;
- *f* is their frequency;
- *x*^last^ characterizes the distribution of the recovery spikes (see Materials and methods for details).

A set of candidate batches $S_{t+1:t+n}$ is thus defined by spanning different values for *m*, *f*, and *x*^last^. Algorithm 3, which is a generalization of Algorithm 2, is used to select the next batch of *n* stimuli $x_{t+1:t+n}^{*}$ in the set $S_{t+1:t+n}$. Every *n* observations, $H_{x_{t+1:t+n}}\left(\Theta |h_{t},Y_{t+1:t+n}\right)$ is computed using *n* iterations of the filter (i.e. Algorithm 1), in order to pick the optimal next batch $x_{t+1:t+n}^{*}$ that minimizes the quantity $H_{x_{t+1:t+n}}\left(\Theta |h_{t},Y_{t+1:t+n}\right)$ (i.e. the posterior entropy over the parameters at time step *t* + *n* given all observations up to time *t*):

$$\begin{array}{c} x_{t+1:t+n}^{*}=\underset{x_{t+1:t+n}\in S_{t+1:t+n}}{\text{arg min}}H_{x_{t+1:t+n}}\left(\Theta |h_{t},Y_{t+1:t+n}\right) \end{array}$$ (20)

Fig 5B shows results from a simulated experiment comparing 4 different protocols:

- The *Deterministic (long)* protocol consists of repetitions of a spike trains made of *m* = 100 stimuli at *f* = 100*Hz* (tetanic phase) followed by 6 recovery pulses at increasing intervals. Therefore, the long protocol consists of *n* = 106 spikes;
- The *Deterministic (short)* protocol is similar to *Deterministic (long)*, except that its tetanic phase only consists of *m* = 20 pulses instead of 100 (hence consisting of *n* = 26 spikes);
- *ESB-BAL (myopic)* optimizes each stimulus, as in Figs 2, 3, and 4;
- *ESB-BAL (batch)* performs batch optimization, as detailed above: every *n* observation, the next batch of *n* stimuli $x_{t+1:t+n}^{*}$ is computed in the set $S_{t+1:t+n}$.

Several observations can be made. Firstly, the *Deterministic (long)* protocol is outperformed by its short counterpart, as the latter yields a larger entropy decrease for a given number of stimulations. Intuitively, this highlights that, apart from the first spikes, the tetanic phase (similarly to the *Constant* stimulation from Fig 2) brings little information about the unknown parameters, and that a few high-frequency stimulations followed by recovery spikes are enough to efficiently probe the synapse. Secondly, batch optimization outperforms myopic optimization, showing that synaptic parameter inference benefits from optimizing not only the next stimulus, but rather the next *n* stimuli of the experiment altogether. Finally, *ESB-BAL (batch)* does not outperform the *Deterministic (short)* protocol, which is likely due to the fact that the latter has been specifically tailored for studying STD and has been defined and optimized through trial and error. Note that the posterior entropy may increase during the tetanic stimulation phase, as explained in Section Particle filtering for synaptic characterization.

**Fig 6:**
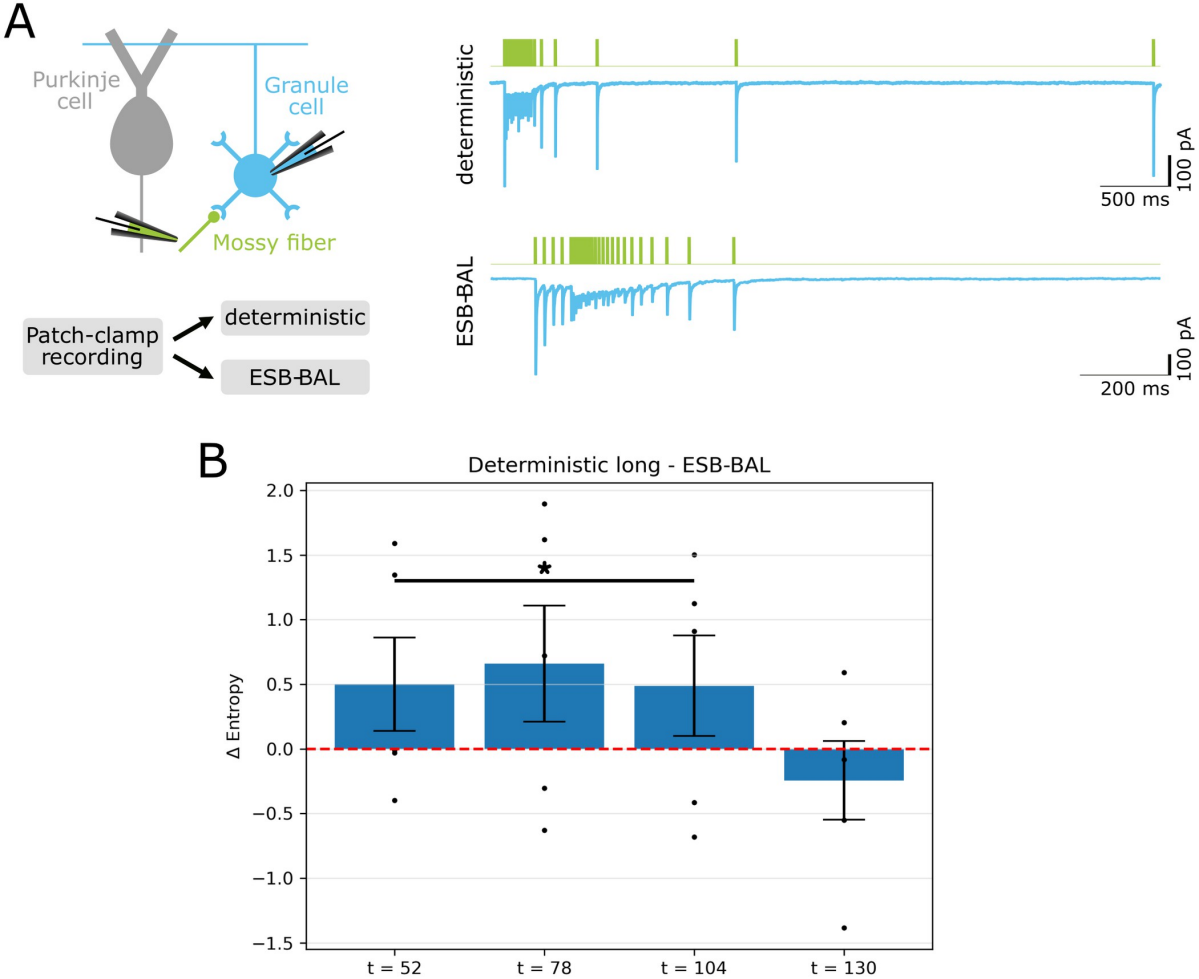
Application to neural recordings. A: Left: Mossy fiber to granule cell synaptic connections from acute cerebellar slices of mice were studied. Each of them was stimulated using successively deterministic protocols and ESB-BAL. Right: examples of postsynaptic current traces recorded from a granule cell upon extracellular mossy fiber stimulation. B: Information gain when comparing the *Deterministic (long)* protocol to ESB-BAL (i.e. the entropy after the deterministic protocol minus the entropy after ESB-BAL) across all studied synapses. A positive value for Δ*Entropy* signifies a lower entropy when using ESB-BAL. Results displayed for different numbers of observations *t*. Test: regression analysis (*p* = 0.0381) comparing entropies after *Deterministic (long)* and ESB-BAL for *t* = 52 to *t* = 104 (see Materials and methods for details).

We validate our method by applying it to EPSC recordings from mossy fiber to granule cell synaptic connections in acute mouse cerebellar slices (Fig 6A), whose depressed nature (S6 Fig) makes them a good match for our theoretical model. Each synapse was stimulated using successively the *Deterministic (long)*, *Deterministic (short)*, and *ESB-BAL (batch)* protocols. For each stimulation protocol, the posterior distribution of the parameters was computed offline using the Metropolis-Hastings algorithm. Fig 6B shows, for different numbers of observations *t*, the information gain when comparing the *Deterministic (long)* protocol to ESB-BAL (i.e. the entropy after the deterministic protocol minus the entropy after ESB-BAL) across all studied synapses: a positive value signifies a lower entropy when using ESB-BAL. Our experimental results are in line with simulations (Fig 5B), as ESB-BAL outperforms the *Deterministic (long)* protocol at the beginning of the experiment (i.e. when *t* is low), but not the *Deterministic (short)* protocol (S7 Fig).

## Discussion

We developed a method called Efficient Sampling-Based Bayesian Active Learning (ESB-BAL) for approximate optimal experimental design. Using particle filtering, ESB-BAL selects the next experimental design to maximize the approximate mutual information between the output of the experiment and the constants of the studied system. To validate it, we apply ESB-BAL to the problem of estimating the constants of a chemical synapse from its postsynaptic currents evoked by presynaptic stimulations. After each new observation, the optimal next stimulation time can be computed using ESB-BAL. Using synthetic data and synaptic whole-cell patch-clamp recordings in cerebellar brain slices, we show that our method is efficient and fast enough to be used in real-time biological experiments and can reduce the uncertainty of inferred parameters.

For illustrative purposes, we applied ESB-BAL to the specific problem of estimating the parameters characterizing a chemical synapse. However, we argue that our framework is sufficiently general and efficient to be applicable to a broad range of systems and domains of research. Especially, our implementation of the Nested Particle Filter can be applied to any state-space system, even time-varying ones. Moreover, as the Nested Particle Filter is robust to time-varying parameters and model uncertainties [13], we believe that our proposed solution will be especially relevant for neurophysiology experiments and for clinical applications, such as the optimization of Deep Brain Stimulation (DBS) for the treatment of Parkinson’s Disease [32, 33].

We expect active learning to be particularly beneficial to neurophysiology experiments involving live cells or subjects. By reducing the number of samples required to obtain a certain result, or by improving the efficiency of information gain, we can reduce the cost of the experiment and the need for animal subjects. A possible negative impact would be that improving the relative efficiency of neurophysiology experiments may lead to a larger field of applications and therefore a larger demand for animal experiments, analogously to Jevons Paradox [34].

Our approach has some room for improvements. An evident drawback of using particle filtering is that it requires a very large number of particles to provide low variance estimates, as the approximation error only decreases with the square root of the number of particles. More generally, the possibility to efficiently apply particle filtering to any statistical model, irrespective of the dimensions for its state variables and observations, is still an open question, as a general theoretical result linking convergence rate and number of states is lacking. The possibility to apply our ESB-BAL framework to other models and experimental settings should first be verified via simulations. Moreover, future theoretical work should focus on obtaining results on the convergence of the estimators when using active learning. When observations are independent and identically distributed (i.i.d.), active learning will give an unbiased estimate of the parameters, whose variance will decrease with the number of observations [29]. Such theoretical results lack for systems with correlated outputs (such as the EPSCs in the studied synapse model), possibly leading to information saturation [35] or biased estimates.

For experimental applications, the amount of time taken to select a new stimulus is of utmost importance. This is especially true for the system studied here, as the selected next ISI should not be shorter than the time it took to compute it. Hence, an approximate, but fast, filtering algorithm is more useful than an exact filtering method, such as the forward algorithm. The particle nature and the recursive structure of the NPF (which makes it computationally efficient) is enabled by approximating the filtering step (which is valid in the limit of a small variance for the jittering kernel), as described in Section The filter: Online computation of the posterior distributions of parameters. This comes at the cost of an approximation error that decreases more slowly with the number of particles than for other particle filtering schemes, such as the SMC² method [36]. Depending on the constraints of the experiment, this trade-off between computational efficiency and accuracy in the estimate the posterior distribution of the parameters *p*(*θ*|*h*_*t*_) can be adjusted by implementing a different filtering scheme.

As an application example, we used ESB-BAL to infer the parameters of an idealized model of chemical synapses, which relies on several assumptions and simplifications. Namely, it assumes that the postsynaptic currents elicited by separate vesicle releases add linearly, so that the final current after *k*_*t*_ vesicle openings is *qk*_*t*_. This assumption disregards possible presynaptic asynchronous release and postsynaptic receptors saturation. There is evidence of linear summation of quanta for the synapses we studied in the experiments [37], but this may not apply to other synapses. Moreover, it also assumes that presynaptic release sites are homogeneous, and share the same values for *p*, *q*, and *τ*_*D*_. Finally, it only accounts for evoked releases and for monosynaptic connections. These assumptions have been widely used in recent models of synaptic transmission, as they allow for tractable analyses while still reflecting the actual observed cellular dynamics [1, 2]. Future experimental work should focus on implementing ESB-BAL for different and more complicated models of a chemical synapse, including for instance short-term facilitation [1, 2, 21, 30, 38] or vesicle content variability [39, 40].

For some recordings in Fig 6B, the benefit of using ESB-BAL instead of a deterministic protocol might seem non-significant. For some synaptic connections (e.g. negative values in Fig 6B), ESB-BAL even yields a higher entropy for the posterior distribution of *θ*. Different explanations can be put forward. Firstly, it is possible that the classically used deterministic protocols (20 stimuli at 100Hz followed by 6 recovery spikes at increasing ISIs, see Fig 6A) are already well informative about the synaptic parameters. For these protocols, the tetanic stimulation phase and the long inter-sweep interval allow to estimate the value of the hidden states *n*_*t*_ and *k*_*t*_ with a high accuracy, which facilitates the estimation of the synaptic parameters [1]. Moreover, the recovery spikes at varying ISIs are known to be more informative about the synapse’s dynamics than a constant stimulation frequency [30]. Secondly, the small benefit of using ESB-BAL for some synaptic connections might be due to a model mismatch issue, as the model defined by Eqs 7 to 10 might not exactly represent the ground-truth mechanisms of the studied synapses. Although mossy fiber to granule cell synapses are believed to be good examples of depressing synapses, our simplified model disregards several aspects of synaptic transmission such as facilitation, postsynaptic saturation, or presynaptic vesicles heterogeneity [37, 41, 42]. These assumptions might explain why ESB-BAL outperforms other methods more consistently in simulations than in real experiments.

An interesting area of future research would be to formulate Optimal Experiment Design as an optimal control problem, using the framework of the Bellman equation [43, 44]. This multi-stage optimization problem could be solved exactly by defining the associated Bellman equation, in which *I*(Θ;*Y*_1:*T*_) is the objective function, current observation *y*_*t*_ is the state, input *x*_*t*_ is the control, and where the optimal policy determines the next input *x*_*t*+1_. This approach would allow to account for the remaining available experimental time. Active Learning has been successfully framed as a Deep Reinforcement Learning problem to select which samples should be used to train Natural Language Processing models when the budget for manual annotations is limited [45]. A policy can be learned on a high-resource language (e.g. English) and then used for another language in which annotated training samples are sparse. In biological experiments and clinical settings, whether a policy can be learned on a cell or a subject and applied to another needs to be investigated.

Bayesian Active Learning is an efficient framework for solving the problem of optimal experiment design for parameters inference. Its goal is, for a given generative model M, to optimize the accuracy of the estimates of the parameters *θ* of M, i.e. to minimize the entropy of the posterior distribution $p(\theta |x_{1:T},y_{1:T},M)$. But it is also possible to extend optimal experiment design to model selection: in this setting, the goal is to maximize the discriminability between competing candidate models, i.e. to minimize the entropy of $p(M|x_{1:T},y_{1:T})$. Different schemes for OED for model selection have been proposed (see [46] for a discussion), but their computational complexity is a major impediment to their concrete applicability. An interesting future application of ESB-BAL would be to extend it to optimal model selection.

Overall, we expect our proposed solution to pave the way towards better estimates of stochastic models in neuroscience, more efficient training in machine learning, and more systematic and automated experimental designs.

When designing an experiment in physiology, or when training a model on data in machine learning, it is common to choose a priori a fixed set of inputs to the studied system. The use of such non-adaptive, non-optimized protocols often leads to a large variance of the estimated parameters, even when using a large number of trials or data points. Bayesian active learning is an efficient method for optimizing these inputs, but exact solutions are often intractable and not applicable to online experiments. Here, we introduce ESB-BAL, a novel framework combining particle filtering, parallel computing, and mean-field theory. ESB-BAL is general and sufficiently efficient to be applied to a wide range of settings. We use it to infer the parameters of a model of synapse: for this specific example, computation time is a critical constraint, since the typical ISI is shorter than 1s, and because several future inputs need to be optimized together. Using synthetic data and neural recordings, we show that our method has the potential to significantly improve the precision and speed of model-based inferences.

## Materials and methods

### Ethics statement

Animals were treated following national and institutional guidelines. The Cantonal Veterinary Office of Zurich approved all experiments (authorization no. ZH009/2020).

### Particle filtering for synaptic characterization

#### Initialisation

Computing the posterior distribution of *θ* firstly implies to specify a prior *p*(*θ*) from which the initial particles ${\left\{\theta_{0}^{i}\right\}}_{1\leq i\leq M_{\text{out}}}$ will be drawn. For simplicity, we consider here uniform priors (as in [2, 21]), although the algorithm readily extends to different choices of prior.

Similarly, initial samples for the hidden states ${\left\{n_{0}^{i,j},k_{0}^{i,j}\right\}}_{1\leq j\leq M_{\text{in}}}$ need to be drawn. For $i\in \{1,...,M_{\text{out}}\}$, $j\in \{1,...,M_{\text{in}}\}$, we define:

-
$n_{0}^{i,j}=N_{i}$ (i.e. all vesicles are supposed to be in the readily-releasable state at the beginning of the simulation);
-
$k_{0}^{i,j}∼\text{Bin}\left(N_{i},p_{i}\right)$ (consistently with Eq 9).

#### Jittering step

The parameters that we wish to infer are supposed to be constant. It is thus impossible to define dynamics of the form $p(\theta_{t+1}^{i}|\theta_{t}^{i})$ for the particles (as opposed to filtering problems aiming at inferring a dynamical hidden state, as for instance in [47]). To avoid particle degeneracy, it is thus necessary to mutate particles using a jittering kernel $\kappa (\theta_{t-1}^{i})$. When particles take continuous values, a classical choice for the jittering kernel is to draw the next particle $\theta_{t}^{i}$ from a Gaussian distribution with mean $\theta_{t-1}^{i}$ and which variance is called the jittering width (see [13] for a detailed discussion). In our implementation, the range of possible values for each parameter is discretized, so that each particle corresponds to a position on the grid of possible parameters values (same implementation as in [2]). The free parameter *α* in our jittering kernel thus corresponds to the probability of moving by one bin:

$$\begin{array}{c} \theta_{t}^{i}=\kappa \left(\theta_{t-1}^{i}\right)=\{\begin{array}{cc} \theta_{t-1}^{i}, & \text{with}\;\text{probability}\;1-\alpha \\ {\tilde{\theta }}_{t-1}^{i}, & \text{with}\;\text{probability}\;\alpha \end{array} \end{array}$$ (21)

where ${\tilde{\theta }}_{t-1}^{i}$ is one (randomly chosen) bin away from $\theta_{t-1}^{i}$.

At each time step, the jittering step tends to increase the entropy of the distribution of the particles (by mutating them). On the other hand, the resampling step (see below) tends to reduce it, by keeping only high-likelihood particles. If the variance of the jittering kernel is sufficiently high to outperform the effect of resampling, the overall entropy of the distribution of the particles will increase (as, for instance, during the tetanic stimulation phase in Fig 5B).

#### Propagation step

Inner particles are drawn based on $n_{t}^{i,j}∼p\left(n_{t}^{i,j}|n_{t-1}^{i,j},k_{t-1}^{i,j},\theta_{t}^{i},x_{t}\right)$ (Eq 10) and $k_{t}^{i,j}∼p\left(k_{t}^{i,j}|n_{t}^{i,j},\theta_{t}^{i}\right)$ (Eq 9).

#### Likelihood computation step

$p(y_{t}|n_{t}^{i,j},k_{t}^{i,j},\theta_{t}^{i})$ is computed according to Eq 8.

#### Resampling step

Particles are resampled by multinomial resampling using the algorithm introduced in [48], which allows to draw a list of sorted numbers in a single step. Alternative resampling schemes can also be implemented. For instance, residual and stratified resampling methods dominate the multinomial one in terms of conditional variance [49]. We found that for artificial data, the stratified method leads to faster convergence of the estimates of *N*, *q*, and *σ*, whereas *p* and *τ*_*D*_ estimates do not show a significant difference of rate of convergence (S8B Fig), but does not significantly improve the information gain (S8A Fig).

**Algorithm 2:** Computation of the optimal next stimulation time for synaptic characterization

**Input**: $S_{t+1}$ (set of candidates *x*_*t*+1_);

**for** *x*_*t*+1_ in $S_{t+1}$ **do**

 Compute $E(Y_{t+1}|x_{1:t+1},{\hat{\theta }}_{t})$ using Eq 23;

 Compute $p(\Theta |h_{t},x_{t+1},Y_{t+1}=E\left(Y_{t+1}|x_{1:t+1},{\hat{\theta }}_{t}\right))$ using Algorithm 1;

 Compute $H_{x_{t+1}}\left(\Theta |h_{t},Y_{t+1}=E\left(Y_{t+1}|x_{1:t+1},{\hat{\theta }}_{t}\right)\right)$ using Eq 16;

**end**

$x_{t+1}^{*}={\text{arg min}}_{x_{t+1}\in S_{t+1}}H_{x_{t+1}}\left(\Theta |h_{t},Y_{t+1}=E\left(Y_{t+1}|x_{1:t+1},{\hat{\theta }}_{t}\right)\right)$

Algorithm 2 is slightly modified in Fig 3 for the “ESB-BAL (exact)” simulations, in which Eq 13 is computed using MC samples instead of the point-based simplifications explained in Eqs 14 and 15. Samples to compute the expectation over *θ* are drawn from the current posterior distribution *p*(*θ*|*h*_*t*_), i.e. by random sampling from the pool of particles ${\left\{\theta_{t}^{i}\right\}}_{i\in \{1,\ldots ,M_{\text{out}}\}}$. For each of these samples, and for each candidate next input *x*_*t*+1_ in $S_{t+1}$:

- In ESB-BAL (MC *θ*): the corresponding value of *y*_*t*+1_ is computed using Eq 23, as in Eq 15;
- In ESB-BAL (MC *θ*, *y*): samples used to compute the expectation over *y*_*t*+1_ are drawn by randomly sampling $n_{t}^{i,j}∼p\left(n_{t}^{i,j}|n_{t-1}^{i,j},k_{t-1}^{i,j},\theta_{t}^{i},x_{t}\right)$ and $k_{t}^{i,j}∼p\left(k_{t}^{i,j}|n_{t}^{i,j},\theta_{t}^{i}\right)$, and using Eq 8.

Unless otherwise specified, all the simulations results were obtained with *M*_out_ = 1024 and *M*_in_ = 256 particles, and were run using a commercially available Nvidia GTX 1080 Ti GPU.

### Input-Output Hidden Markov Model (IO-HMM) of synaptic transmission

In line with previous works (and especially with [1, 2]), we used a simplified Input-Output Hidden Markov Model (IO-HMM) to describe the synapse (as explained in Section The system: A binomial model of neurotransmitter release). Its specificity, compared to classical HMMs, is that *N* is considered as a parameter to be optimized together with the other ones. Besides, the value of *N* characterizes the range of values that the hidden states can have, as 0 ≤ *k*_*t*_ ≤ *n*_*t*_ ≤ *N*. De facto, the exact value of *N* for the studied synapse is unknown, can take a broad range of possible values [1], and needs to be inferred from the observations. Different approaches have been used in previous works:

- In [1], the authors fixed the value for *N* and then estimated the other parameters using the Expectation-Maximization (EM) algorithm. The procedure is repeated for different values of *N* ranging from 1 to 100, and the set of parameters yielding the highest likelihood is selected as the maximum likelihood estimator. In our setting, this would be equivalent to running several instances of ESB-BAL in parallel for different values of *N*, which would significantly increase the computational load.
- In [2], the authors used the Metropolis-Hastings algorithm to compute the posterior distribution of the parameters, including *N*. In our case, we also jointly optimize all parameters, including *N*. This means that, due to the jittering kernel, $N_{t}^{i}$ might change from time to time. As a consequence, we introduce the ReLU function to keep the value of $n_{t}^{i,j}$ positive in Algorithm 1 (where $N_{t}^{i}$ and $p_{t}^{i}$ refer to the values of *N* and *p* in particle $\theta_{t}^{i}$):

$n_{t}^{i,j}\leftarrow ReLU\left(N_{t}^{i}-n_{t-1}^{i,j}+k_{t-1}^{i,j}\right)$

$k^{i,j}\leftarrow rand(\text{Bin}\left(n_{t}^{i,j},\pi \left(x_{t}\right)\right)$

$n_{t}^{i,j}\leftarrow N_{t}^{i}-n_{t}^{i,j}+k^{i,j}$

$k_{t}^{i,j}\leftarrow rand\left(\text{Bin}\left(n_{t}^{i,j},p_{t}^{i}\right)\right)$

### Mean-field approximation of vesicle dynamics

Our synapse model, as defined by Eqs 7 to 10, is a Hidden Markov Model with observations *y*_*t*_ and hidden states *z*_*t*_ = (*n*_*t*_, *k*_*t*_). The predictive distribution *p*(*y*_*t*+1_|*h*_*t*_, *x*_*t*+1_, *θ*) used in Eq 4 can be computed using the forward algorithm: however, the algorithmic complexity of this exact filtering procedure, which scales with *N*^4^, makes it impractical for closed-loop applications. Here, we suggest that computation can be massively simplified by using a mean-field approximation of vesicle dynamics: the analytical mean of hidden and observed variables can be computed using recursive formulæ [46].

Let *r*_*t*_ ∈ [0, 1] denote the average fraction of release-competent vesicles at the moment of spike *t*. Its values, given *θ* = [*N*, *p*, *q*, *σ*, *τ*_*D*_] and *x*_1:*t*_, can be iteratively computed (see [1], Eq (7)) from the equations of the Tsodyks-Markram model [20]:

$$\begin{array}{c} r_{t}=1-\left(1-\left(1-p\right)r_{t-1}\right)\text{exp}(-\frac{x_{t}}{\tau_{D}}) \end{array}$$ (22)

with *r*_1_ = 1. It follows that the expected value of the EPSC after spike *t* is

$$\begin{array}{c} E\left(Y_{t}|x_{1:t},\theta \right)=r_{t}Npq \end{array}$$ (23)

It is similarly possible to compute the variance of the observation, which is used in S6 Fig. One can note that the variance of the number of available vesicles *n*_*t*_ conditioned on the history of previous activations *x*_1:*t*_ and on the parameter values *θ* can be computed similarly using the law of total variance:

$$\begin{array}{c} \text{Var}\left(n_{t}|x_{1:t},\theta \right)=E\left(\text{Var}\left(n_{t}|n_{t-1},k_{t-1},x_{1:t},\theta \right)\right)+\text{Var}\left(E\left(n_{t}|n_{t-1},k_{t-1},x_{1:t},\theta \right)\right) \end{array}$$ (24)

Since *n*_*t*_ = *n*_*t*−1_ − *k*_*t*−1_ + *v*_*t*_ with *v*_*t*_ ∼ Bin(*N* − *n*_*t*−1_ + *k*_*t*−1_, *π*(*x*_*t*_)) (see Eq 10), it follows that

$$\begin{array}{c} \text{Var}\left(n_{t}|x_{1:t},\theta \right)=\pi \left(x_{t}\right)\left(1-\pi \left(x_{t}\right)\right)N\left(1-r_{t-1}+pr_{t-1}\right)+\\ {\left(1-\pi \left(x_{t}\right)\right)}^{2}\text{Var}\left(n_{t-1}-k_{t-1}|x_{1:t-1},\theta \right) \end{array}$$ (25)

Finally, by noting that (*n*_*t*_ − *k*_*t*_)|*n*_*t*_ ∼ Bin(*n*_*t*_, 1 − *p*) and using again the law of total variance to compute

$$\begin{array}{c} \text{Var}\left(n_{t-1}-k_{t-1}|x_{1:t-1},\theta \right)=E\left(\text{Var}\left(n_{t-1}-k_{t-1}|n_{t-1},x_{1:t-1},\theta \right)\right)+\\ \text{Var}(E\left(n_{t-1}-k_{t-1}|n_{t-1},x_{1:t-1},\theta \right)) \end{array}$$ (26)

we obtain

$$\begin{array}{c} \text{Var}\left(Y_{t}|x_{1:t},\theta \right)=\sigma^{2}+q^{2}\left(Nr_{t}p\left(1-p\right)+\text{Var}\left(n_{t-1}|x_{1:t-1},\theta \right)p^{2}\right) \end{array}$$ (27)

Eqs 23 and 27 are respectively used to compute the expected value of *y*_*t*_ given *θ* and *x*_1:*t*_ (which is used in the point-based approximation of Eq 15) and its variance (which is used to verify the goodness of fit of our model to recorded EPSCs in S6 Fig).

### Batch optimization

Each candidate batch of *n* stimulation times in $S_{t+1:t+n}$ (Fig 5A) is described by 3 parameters:

- *m* < *n*: the number of tetanic stimulations [-];
- *f*: the frequency of the tetanic stimulations [Hz];
- *x*^last^: the time interval before the final recovery spike [s].

A train of *n* stimulations is thus composed of *m* tetanic stimulations at a frequency *f*, followed by *n* − *m* recovery spikes with increasing inter-spike intervals $\frac{x^{\text{last}}}{n-m},\frac{x^{\text{last}}}{n-m-1},\ldots ,\frac{x^{\text{last}}}{2},x^{\text{last}}$. The following values were used during experiments (Fig 6): *n* = 26, *m* ∈ [5, 10, 15, 20], *f* ∈ [25*Hz*, 50*Hz*, 100*Hz*, 200*Hz*], *x*^last^ ∈ [0.1*s*, 0.5*s*, 1.0*s*, 2.0*s*].

These 4 possible values for each parameter *m*, *f*, and *x*^last^ yield 64 different combinations: $S_{t+1:t+n}$ thus consists of 64 different candidate batches, from which Algorithm 3 picks the next optimal one.

**Algorithm 3:** Computation of the optimal next batch of ISIs

**Input**: $S_{t+1:t+n}$ (set of candidates *x*_*t*+1:*t*+*n*_);

**for** *x*_*t*+1:*t*+*n*_ *in* $S_{t+1:t+n}$ **do**

 Compute $H_{x_{t+1:t+n}}\left(\Theta |h_{t},Y_{t+1:t+n}\right)$ using Algorithm 1;

**end**

$x_{t+1:t+n}^{*}={\text{arg min}}_{x_{t+1:t+n}\in S_{t+1:t+n}}H_{x_{t+1:t+n}}\left(\Theta |h_{t},Y_{t+1:t+n}\right)$

### Electrophysiological recordings

Experiments were performed in adult (> 1-month-old) male and female C57BL/6J mice (Janvier Labs, France). Animals were housed in groups of 3–5 in standard cages on a 12h-light/12h-dark cycle with food and water ad libitum. Mice were sacrificed by rapid decapitation after isoflurane anesthesia. The cerebellar vermis was removed quickly and mounted in a chamber filled with cooled extracellular solution. 300-μm thick parasagittal slices were cut using a Leica VT1200S vibratome (Leica Microsystems, Germany), transferred to an incubation chamber at 35 °C for 30 minutes, and then stored at room temperature until experiments.

The extracellular solution (artificial cerebrospinal fluid, ACSF) for slice cutting and storage contained (in mM): 125 NaCl, 25 NaHCO3, 20 D-glucose, 2.5 KCl, 2 CaCl2, 1.25 NaH2PO4, 1 MgCl2, bubbled with 95% O2 and 5% CO2. Slices were visualized using an upright microscope with a 60×, 1 NA water-immersion objective, infrared optics, and differential interference contrast (Scientifica, UK). The recording chamber was continuously perfused with ACSF supplemented with 10 μM D-APV, 10 μM bicuculline, and 1 μM strychnine. Experiments were performed at room temperature (21–25 °C). Patch pipettes (open-tip resistances of 3–8 MΩ) were filled with solution containing (in mM): 150 K-D-gluconate, 10 NaCl, 10 HEPES, 3 MgATP, 0.3 NaGTP, 0.05 ethyleneglycol-bis(2-aminoethylether)-N,N,N’,N’-tetraacetic acid (EGTA), pH adjusted to 7.3 using KOH.

Voltage-clamp recordings were done using a HEKA EPC10 amplifier controlled via Patchmaster software (HEKA Elektronik GmbH, Germany) essentially as described in [50]. Voltages were corrected for a liquid junction potential of +13 mV. Extracellular mossy fiber stimulation was performed using square voltage pulses (duration, 150 μs) generated by a stimulus isolation unit (ISO-STIM 01B, NPI) and applied through an ACSF-filled pipette. The pipette was moved over the slice surface close to the postsynaptic cell while applying voltage pulses until excitatory postsynaptic currents (EPSCs) could be evoked reliably. Care was taken to stimulate single mossy fiber inputs. EPSCs were recorded at a holding potential of –80 mV; data were low-pass filtered at 2.9 kHz and digitized at 20–50 kHz. Train stimulation protocols comprised bouts of 20 or 100 MF stimulations at 100 Hz, followed by single pulses to monitor recovery from short-term depression (intervals: 25 ms, 50 ms, 100 ms, 300 ms, 1 s, 3 s). The interval between subsequent train recordings was at least 30 s. For OED experiments, custom protocols were generated online as file templates for use with Patchmaster. EPSCs were quantified as peak amplitudes from a 300-μs baseline before onset.

To facilitate the definition of the range of possible values for parameters *q* and *σ* (and especially to avoid running an experiment with too narrow ranges), recorded EPSC amplitudes were normalized by dividing them by their maximum value. Analyses are thus performed by assuming *q* ∈ [0, 1] and *σ* ∈ [0, 1].

Data were collected from 12 synaptic connections, each of them being successively stimulated with the *Deterministic (long)*, *Deterministic (short)*, and ESB-BAL protocols. To accurately compare the effect of ESB-BAL to this of deterministic protocols, it is important to ensure that the synapse (and especially the strength of the synaptic connection) has not changed between successive protocols (as synaptic strength can rapidly change in between stimulation protocols due to synaptic plasticity). For each protocol, the mean value of the first EPSC of each batch is computed, and the relative difference of this value for a pair of protocols is used to assess the drift between these protocols. For a given synapse, pairs of protocols (i.e. *Deterministic (long)* vs. ESB-BAL and *Deterministic (short)* vs. ESB-BAL) for which this drift exceeded 10% were discarded, hence effectively leaving 5 pairs of protocols in Fig 6B and 7 in S7 Fig.

### EPSC quantification

Data were sampled at 20kHz. To quantify EPSC peak amplitudes, we first used a boxcar smoothing of raw data with a 5-point (i.e. 250*μs*) window. EPSC peak amplitude was then defined as the difference between the mean of a 0.4-ms baseline after the stimulation artifact and the minimum in the following 4-ms time window. Due to the synaptic delay, the EPSC starts about 800*μs* to 1*ms* after the stimulation, thus allowing to compute the baseline between the stimulation artifact and the EPSC onset. This has advantages in high-frequency stimulation trains, when the preceding EPSC may not have decayed to baseline.

### Statistical testing

Linear regression is used to measure the effect of ESB-BAL compared to a deterministic protocol. For data shown in Fig 6B, entropy (dependent variable) is regressed against the categorical variable corresponding to the used protocol (ESB-BAL or deterministic). t-test is then performed on the fitted slope coefficient.

## Supporting information

S1 Fig Examples of posteriors obtained using the filter (Algorithm 1). Upper left panel: train of synthetic EPSCs generated from the model described in Section The system: A binomial model of neurotransmitter release. Other panels: posterior distributions of the parameters after 230 stimulations. Ground-truth values used to generate the EPSCs are displayed as red vertical lines.
(TIFF)

S2 FigAverage final entropy decrease (i.e. information gain) after 200 observations using the *Constant* (top), *Uniform* (middle), or *Exponential* (bottom) protocol, for different values of their hyperparameters.Ground truth parameters used are N* = 7, p* = 0.6, q* = 1 pA, σ* = 0.2 pA, and $\tau_{D}^{*}=0.25$s [2]. Vertical red lines indicate the ground truth value $\tau_{D}^{*}=0.25$s used for simulations. Optimal values for *x*_*t*_, *x*^max^, and *τ* are used in Fig 2. For the hyperparameter *x*^*max*^, values up to 2s are spanned to ensure that the mean of the *Uniform* distribution will span values from 0 to 1s.(TIFF)

S3 Fig Comparison of ESB-BAL to an optimized non-parametric fixed design. In previous analyses, our adaptative design (ESB-BAL) was compared to parametric fixed designs (Constant, Uniform, and Exponential). Although intuitive, these parametric fixed protocols may not accurately represent the most informative fixed design. The best fixed ISI distribution can be computed non-parametrically by randomly drawing ISIs from the list of previous ISIs computed via ESB-BAL. We thus obtain a fixed and non-parametric optimized design. Results show that this best fixed design (purple) performs similarly to the best exponential design (green) and is still outperformed by ESB-BAL (black).
(TIFF)

S4 FigSame setting as in Fig 2 but for ground truth parameters N* = 10, p* = 0.85, q* = 1 pA, σ* = 0.2 pA, and $\tau_{D}^{*}=0.2$s.(TIFF)

S5 Fig Same setting as in Fig 2 but when optimizing solely for the marginal posterior distribution of *τ*_*D*_. (TIFF)

S6 Fig The binomial model provides a good fit to recorded EPSCs. Postsynaptic responses to presynaptic stimulations were recorded in mossy fiber to granule cell synaptic connections from acute cerebellar slices of mice. EPSCs amplitudes are computed from raw traces, as detailed in Materials and methods. In this trace example, the presynaptic axon was stimulated using repetitions of a deterministic train of spikes composed of 20 stimulations at 100Hz (tetanic stimulation) followed by 6 recovery spikes at increasing ISIs. This trace illustrates the short-term depression of the studied synapses, visible in the lower EPSCs amplitudes following the first spike in the tetanic phase, and in the increasing amplitudes during the recovery phase. The goodness of fit of the binomial model (described in Section The system: A binomial model of neurotransmitter release) is assessed by comparing its prediction to recorded EPSCs. For a given synapse, we first obtain maximum a-posteriori estimates of its parameters *θ* using Metropolis-Hastings samples (as in [2]). Second, at each time step *t*, the value of the expected EPSC *y*_*t*_ and its variance given *θ* and *x*_1:*t*_ can be computed using Eqs 23 and 27. This prediction from the model (mean: orange solid line; shaded area: 3 standard deviations) can then be compared to actual recordings (blue solid line).
(TIF)

S7 Fig Same setting as in Fig 6B but comparing *Deterministic (short)* and *ESB-BAL (batch)*. (TIF)

S8 Fig Comparison of the effect of Multinomial and Stratified resampling. Same setting as in Fig 2. Simulations used either the Multinomial or Stratified schemes for particles resampling (see Section Particle filtering for synaptic characterization). Although the Stratified resampling scheme improves the convergence of the parameters (B), it does not significantly improve the information gain (A).
(TIFF)
