# Supplementary figures and images for: Efficient sampling-based Bayesian Active Learning for synaptic characterization
Source: PLoS Comput Biol. 2023 Aug 21;19(8):e1011342. doi: 10.1371/journal.pcbi.1011342 (PMC10470935; doi:10.1371/journal.pcbi.1011342)

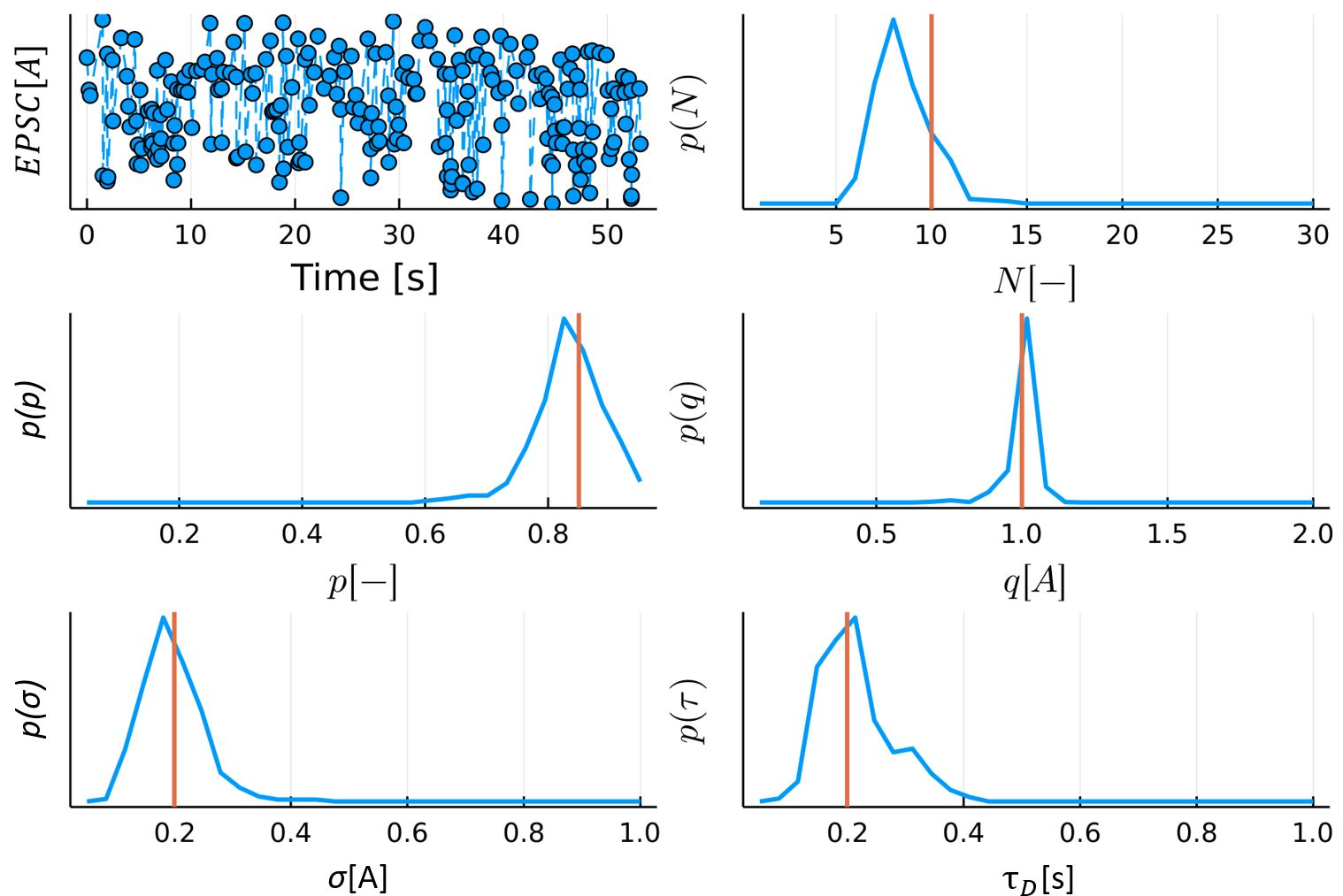

Supplement: S1 Fig — Upper left panel: train of synthetic EPSCs generated from the model described in Section The system: A binomial model of neurotransmitter release. Other panels: posterior distributions of the parameters after 230 stimulations. Ground-truth values used to generate the EPSCs are displayed as red vertical lines. (TIFF) [file pcbi.1011342.s001.tiff]

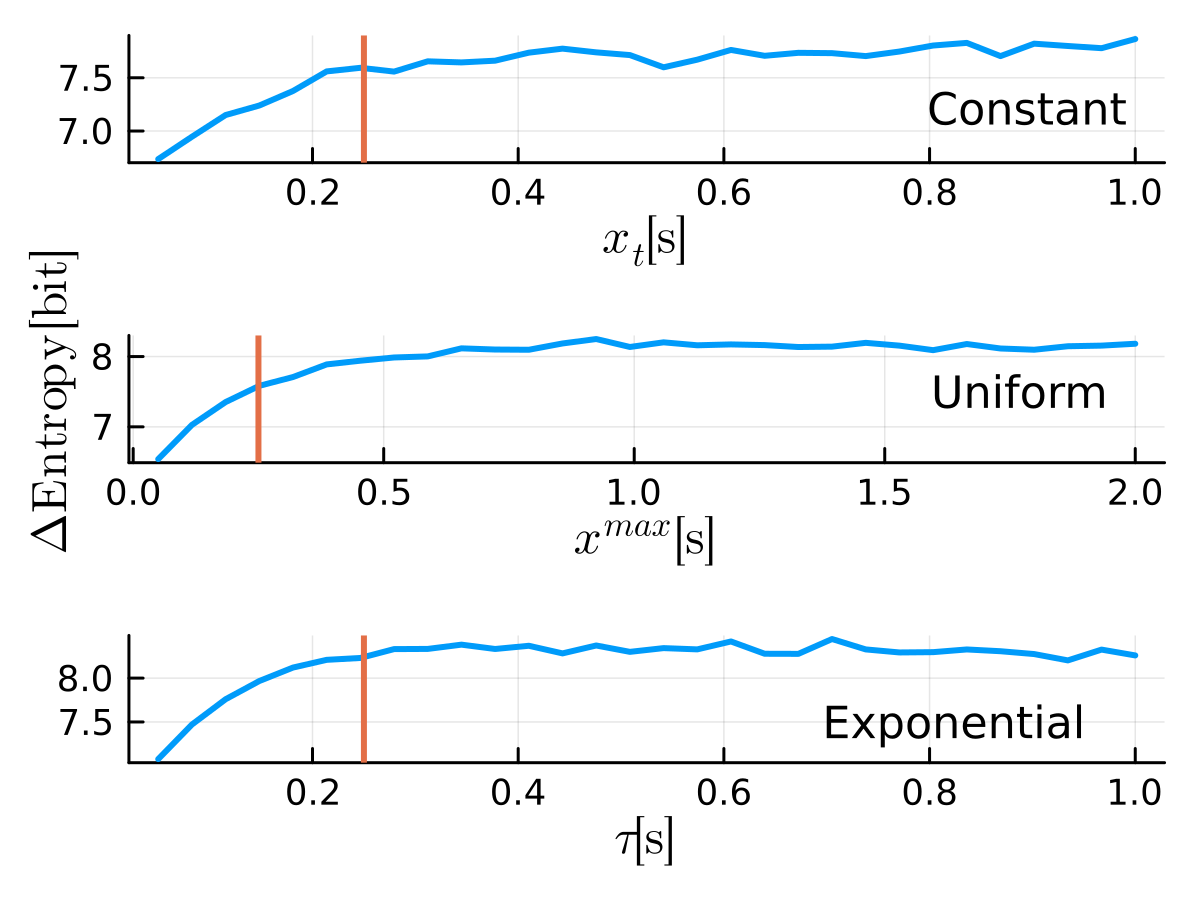

Supplement: S2 Fig — Ground truth parameters used are N* = 7, p* = 0.6, q* = 1 pA, σ* = 0.2 pA, and τD*=0.25s [2]. Vertical red lines indicate the ground truth value τD*=0.25s used for simulations. Optimal values for xt, xmax, and τ are used in Fig 2. For the hyperparameter xmax, values up to 2s are spanned to ensure that the mean of the Uniform distribution will span values from 0 to 1s. (TIFF) [file pcbi.1011342.s002.tiff]

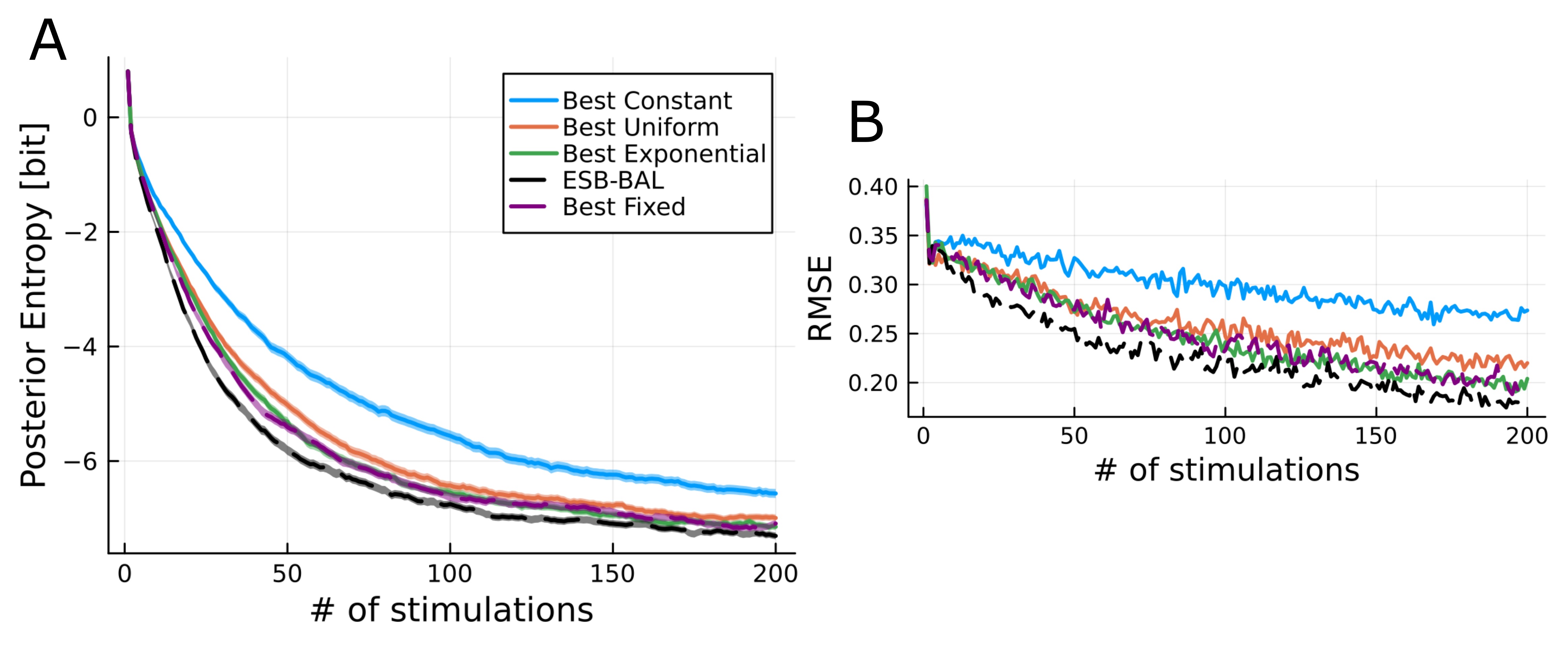

Supplement: S3 Fig — In previous analyses, our adaptative design (ESB-BAL) was compared to parametric fixed designs (Constant, Uniform, and Exponential). Although intuitive, these parametric fixed protocols may not accurately represent the most informative fixed design. The best fixed ISI distribution can be computed non-parametrically by randomly drawing ISIs from the list of previous ISIs computed via ESB-BAL. We thus obtain a fixed and non-parametric optimized design. Results show that this best fixed design (purple) performs similarly to the best exponential design (green) and is still outperformed by ESB-BAL (black). (TIFF) [file pcbi.1011342.s003.tiff]

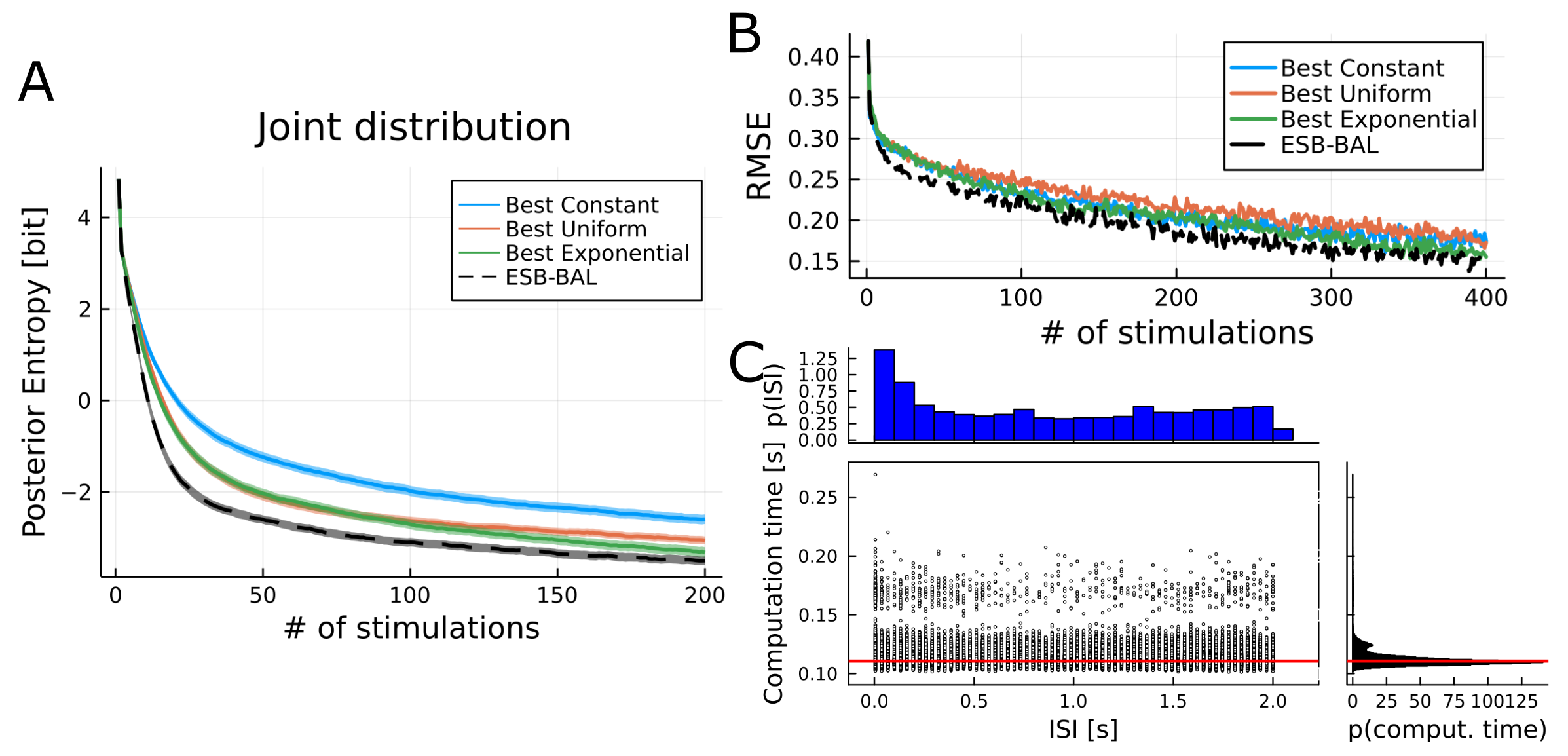

Supplement: S4 Fig — (TIFF) [file pcbi.1011342.s004.tiff]

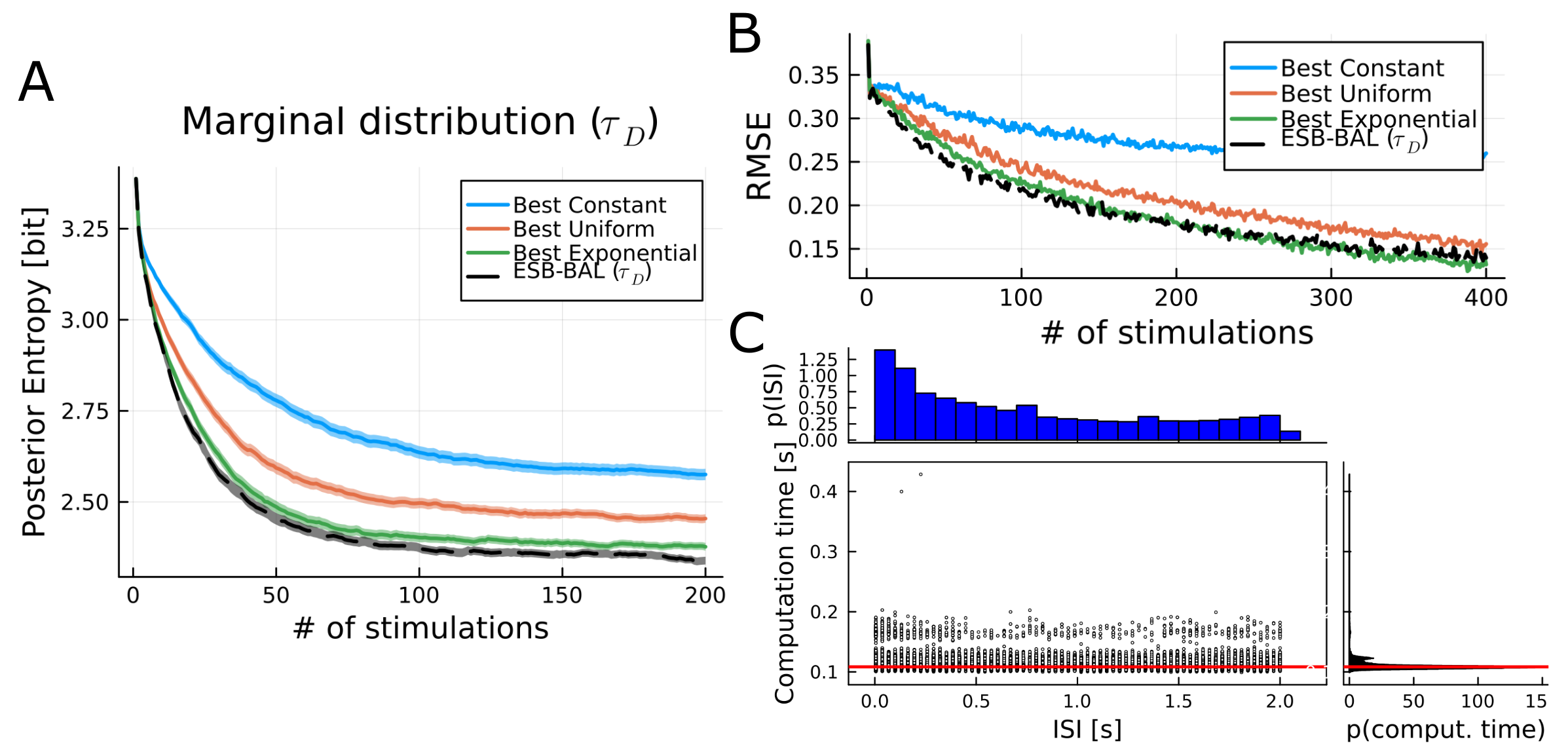

Supplement: S5 Fig — (TIFF) [file pcbi.1011342.s005.tiff]

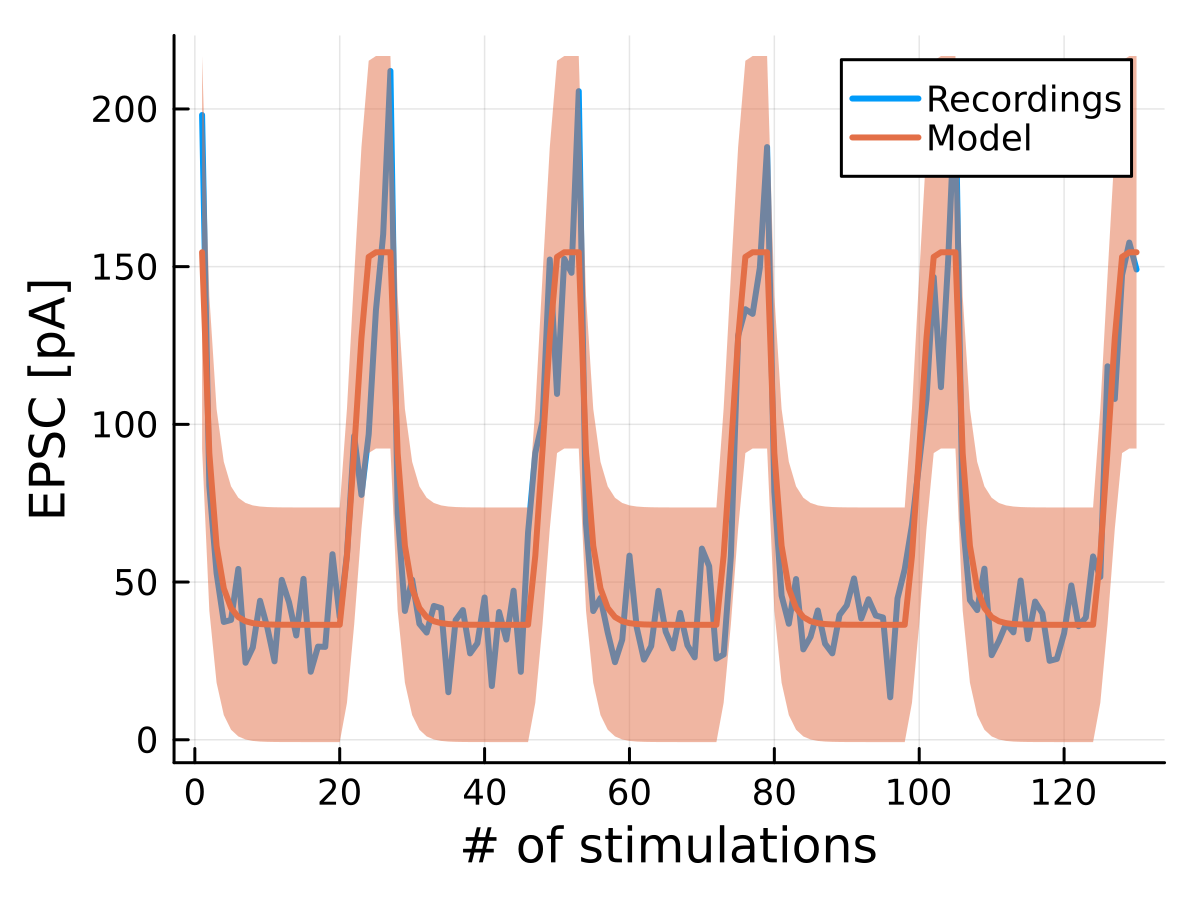

Supplement: S6 Fig — Postsynaptic responses to presynaptic stimulations were recorded in mossy fiber to granule cell synaptic connections from acute cerebellar slices of mice. EPSCs amplitudes are computed from raw traces, as detailed in Materials and methods. In this trace example, the presynaptic axon was stimulated using repetitions of a deterministic train of spikes composed of 20 stimulations at 100Hz (tetanic stimulation) followed by 6 recovery spikes at increasing ISIs. This trace illustrates the short-term depression of the studied synapses, visible in the lower EPSCs amplitudes following the first spike in the tetanic phase, and in the increasing amplitudes during the recovery phase. The goodness of fit of the binomial model (described in Section The system: A binomial model of neurotransmitter release) is assessed by comparing its prediction to recorded EPSCs. For a given synapse, we first obtain maximum a-posteriori estimates of its parameters θ using Metropolis-Hastings samples (as in [2]). Second, at each time step t, the value of the expected EPSC yt and its variance given θ and x1:t can be computed using Eqs 23 and 27. This prediction from the model (mean: orange solid line; shaded area: 3 standard deviations) can then be compared to actual recordings (blue solid line). (TIF) [file pcbi.1011342.s006.tif]

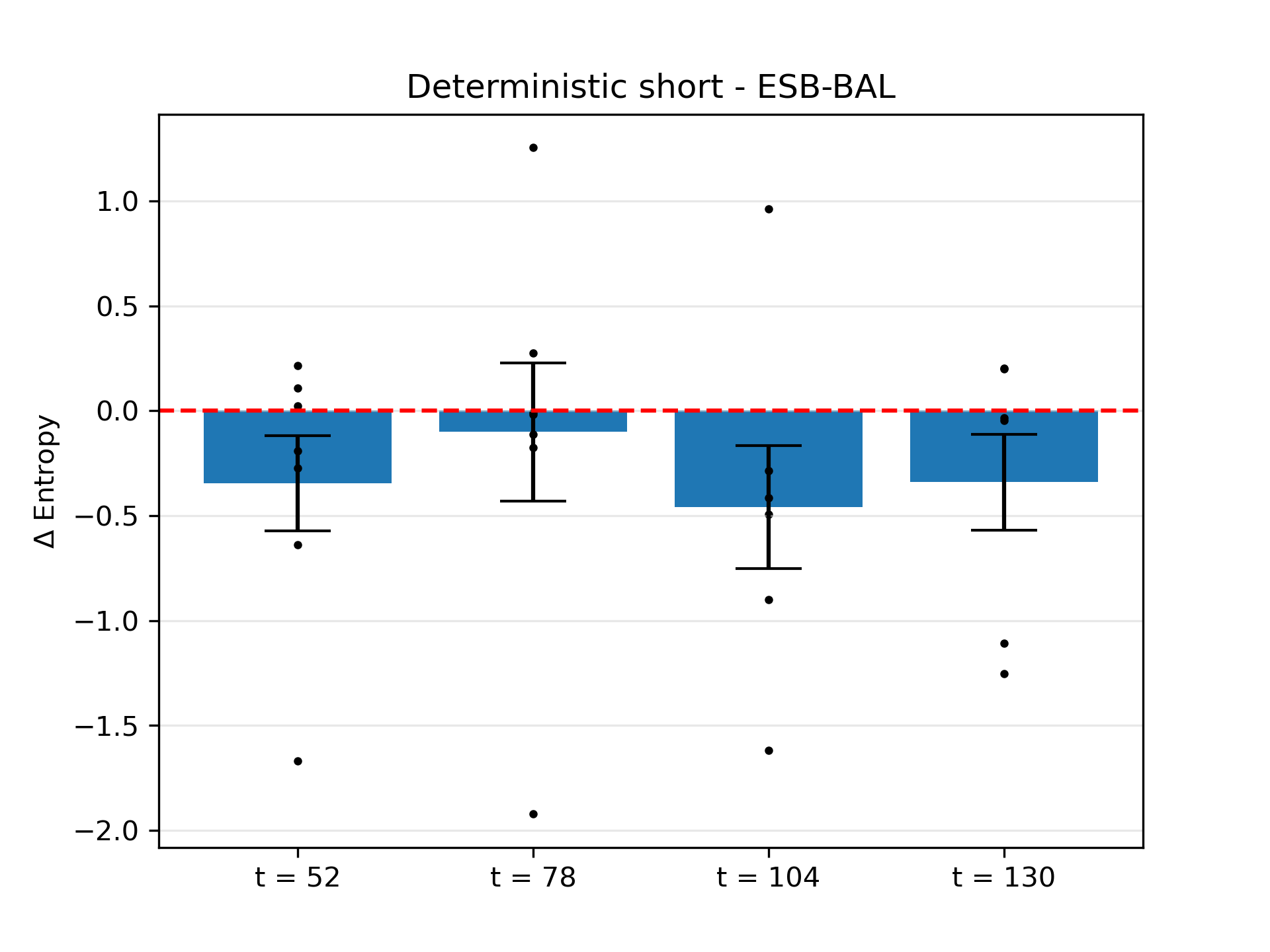

Supplement: S7 Fig — (TIF) [file pcbi.1011342.s007.tif]

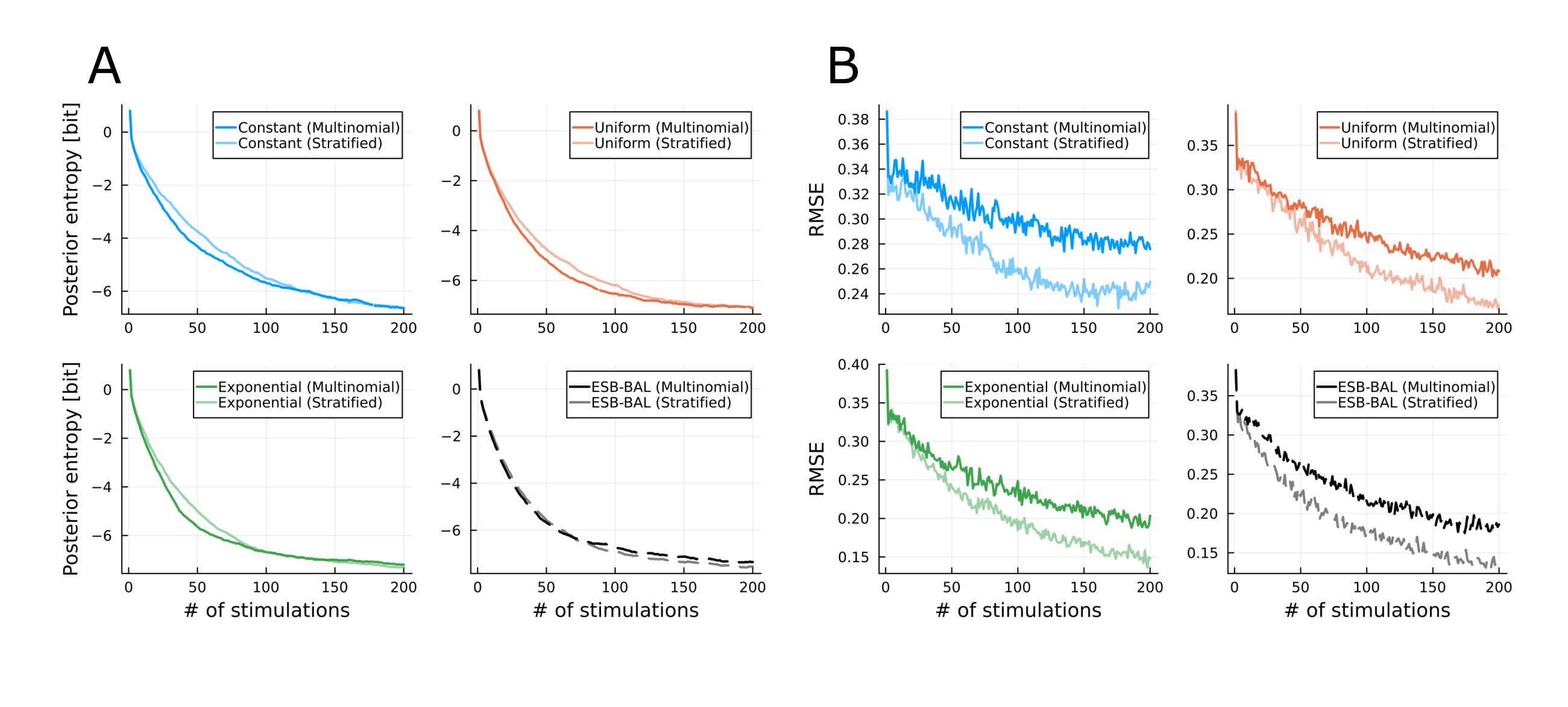

Supplement: S8 Fig — Same setting as in Fig 2. Simulations used either the Multinomial or Stratified schemes for particles resampling (see Section Particle filtering for synaptic characterization). Although the Stratified resampling scheme improves the convergence of the parameters (B), it does not significantly improve the information gain (A). (TIFF) [file pcbi.1011342.s008.tiff]
